# YOLOv8 forestry pest recognition based on improved re-parametric convolution

**DOI:** 10.3389/fpls.2025.1552853

**Published:** 2025-03-11

**Authors:** Lina Zhang, Shengpeng Yu, Bo Yang, Shuai Zhao, Ziyi Huang, Zhiyin Yang, Helong Yu

**Affiliations:** ^1^ College of Information Technology, Jilin Agricultural University, Changchun, China; ^2^ School of Information Engineering, Changchun University of Finance and Economics, Changchun, China

**Keywords:** model pruning, pest identification, YOLOv8, HGNetv2, heavy parameter lightweight convolution

## Abstract

**Introduction:**

The ecological and economic impacts of forest pests have intensified, particularly in remote areas. Traditional pest detection methods are often inefficient and inaccurate in complex environments, posing significant challenges for effective pest management. Enhancing the efficiency and accuracy of pest detection under resource-limited conditions has thus become a critical issue. This study aims to address these challenges by proposing an improved lightweight forestry pest detection algorithm, RSD-YOLOv8, based on YOLOv8.

**Methods:**

To improve the performance of pest detection, we introduced several modifications to the YOLOv8 architecture. First, we proposed RepLightConv to replace conventional convolution in HGNetV2, forming the Rep-HGNetV2 backbone, which significantly reduces the number of model parameters. Additionally, the neck of the model was enhanced by integrating a slim-neck structure and adding a Dyhead module before the output layer. Further optimization was achieved through model pruning, which contributed to additional lightweighting of the model. These improvements were designed to balance detection accuracy with computational efficiency, particularly for deployment in resource-constrained environments.

**Results:**

The experimental results demonstrate the effectiveness of the proposed RSD-YOLOv8 model. The model achieved a Map@0.5:0.95(%) of 88.6%, representing a 4.2% improvement over the original YOLOv8 model. Furthermore, the number of parameters was reduced by approximately 36%, the number of operations decreased by 36%, and the model size was reduced by 33%. These improvements indicate that the RSD-YOLOv8 model not only enhances detection accuracy but also significantly reduces computational burden and resource consumption.

**Discussion:**

The lightweight technology and architectural improvements introduced in this study have proven effective in enhancing pest detection accuracy while minimizing resource requirements. The RSD-YOLOv8 model's ability to operate efficiently in remote areas with limited resources makes it highly practical for real-world applications. This advancement holds positive implications for agroforestry ecology and supports the broader goals of intelligent and sustainable development. Future work could explore further optimization techniques and the application of this model to other domains requiring lightweight and accurate detection systems.

## 1 Introduction

With the rapid spread of plantation forests worldwide in recent years (Bongaarts, 2019), the problem of forestry pests has become more and more prominent, and there has been a significant increase in the speed and range of their spread, both native and invasive pests (Yang et al., 2014). Large-scale outbreaks of pests have caused severe economic losses and ecological damage to forest resources (Choi and Park, 2019). Therefore, it has become particularly urgent and necessary to strengthen the effective detection and monitoring of forestry pests (Zhao et al., 2023).

Traditional identification of forestry pests has relied mainly on a limited number of environmentalists and entomologists (Al-Hiary et al., 2011). This process usually involves manual and visual inspection of insects based on their physical characteristics (e.g., wings, antennae, mouthparts, and legs). This approach has obvious drawbacks due to the diversity of pests and the subtle differences between species. With the continuous advancement of machine learning and computer vision techniques (Dong et al., 2024), the automatic identification of pests has attracted much attention. Deep learning has revolutionized the field of image processing, surpassing traditional methods that require human intervention, such as visual recognition and manual selection (Liu and Wang, 2021). Deep learning has achieved considerable success in target detection and has become a mainstream approach for detection tasks. YOLO (Redmon et al., 2016) (You Only Look Once), a target detection model, was first proposed in 2015. The core feature of this model is its implementation of real-time target detection, a major technological breakthrough at the time. The YOLO model treats the target detection task as a unified, end-to-end deep learning process and no longer repurposes classifiers for the detection task as compared to SSD (Liu et al., 2016) and Faster R-CNN (Ren et al., 2016). YOLO achieves this goal by dividing the entire image into a fixed number of grids and simultaneously predicting the class and location of the target within each grid, achieving significant improvements in speed and efficiency.

Although the YOLO series models have made significant progress in the field of object detection, they still face some challenges in forestry pest detection tasks. For example, complex background environments can interfere with the detection accuracy of the model; The diversity of pest morphology, such as differences in size, color, and shape among different species of pests, increases the difficulty of identification; Higher demands have been placed on the computational complexity and size of the model.

The main contents of this study are as follows:

1. redesign HGNetV2: RepLightConv is proposed to replace the conventional convolution in the original HGNetV2, and the backbone network is optimized by using the reparametrized convolution, which reduces the number of parameters and computation and improves the detection speed.
2. Introducing slim-neck structure: replacing C2f and standard convolution in the YOLOv8 neck layer. It significantly reduces the computational complexity and network architecture while maintaining the high accuracy performance of the model.
3. Introduction of Dyhead detection head: improves the model’s sensitivity to key feature extraction in the pest detection task and, at the same time, enhances the model’s adaptability to different target features and transformations, which effectively improves the detection accuracy of pest targets in different background environments.
4. Model Pruning Optimization Model: A lighter detection model is obtained by introducing layer-based adaptive amplitude pruning (Lamp).

## 2 Related work

Researchers have recently proposed various improved deep learning-based models for pest detection tasks in recent years. **Table 1** shows the research on pests and diseases in the YOLO series in recent years. The YOLOv8 model has been improved to maintain accuracy while reducing computational costs (Jiang and Chen, 2024). however, their study was limited by a small dataset (2,183 samples) and a uniform experimental background (white color) with only seven pest categories, which made it complex and dynamic detection environments challenging. The Skip DETR was proposed by (Liu et al., 2023). effectively improves feature fusion between different network layers by introducing skip connections and spatial pyramid pooling layers, thereby significantly enhancing detection accuracy. However, its high computational complexity (36.8M parameters) limits its practical application in resource-constrained remote areas. Zhang et al. (2023a) proposed an improved YOLOv8 model, which effectively solved the problem of gradient vanishing when training the algorithm on the IP102 (Wu et al., 2019) dataset. An enhanced YOLOv5 model tailored for rice pests was introduced by (Yu and Zhang, 2023), which significantly reduces the computational complexity and presents a large-scale feature extraction layer to improve the detection of small and medium-sized pest targets in rice.

**Table 1:** Comparison table of pest identification research.

References	Dataset size	Recognition algorithm	Map@0.5:0.95(%)	Params (M)	Classes
Jiang and Chen, 2024	2138	YOLOv8	82.3	\	7
Liu et al., 2023	7163	DETR	77.0	36.8M	31
Zhang et al., 2023a	19000	YOLOv8	39.4	25.8M	102
Yu and Zhang, 2023	2120	YOLOv5	66.2	1.94M	9
Cheng et al., 2022	3000	YOLOv3	82.9(Map@0.5)	5M	15
Li et al., 2022a	4418	YOLOv5	96.63(Map@0.5)	\	10
Xiang et al., 2023	576	YOLOv5	74.3	6.0M	28
Yang et al., 2023	4532	YOLOv7	51.2	33.4M	13
Wen et al., 2022	25378	YOLOv4	69.5(Map@0.5)	\	24
Checola et al., 2024	615	YOLOv8	66.0	\	1

Further advances by (Cheng et al., 2022) include, a lightweight YOLOv3-based crop pest detection method that reduces the number of parameters and computations required. Li et al. (2022a) developed YOLOL-JD, a jute pest identification model with an average accuracy (Map50) of 96.63%. Xiang et al. (2023) presented Yolo Pest, a simplified model structure designed to minimize the loss of pest traits for small targets without compromising the detection of normal-sized targets. Yang et al. (2023) introduced Yolo for maize, an innovative YOLOv7-based maize pest detection method enhanced by CSPResNeXt-50 and VoVGSCSP modules, which improves the accuracy and speed of network detection while reducing computational requirements. The Pest YOLO (Wen et al., 2022), which minimizes the parameter counts while maintaining the detection performance to mitigate the waste of computational resources and inefficiency caused by over-parameterization. In addition, a fast and lightweight passion fruit pest detection algorithm based on the improved YOLOv5 was developed, which significantly improves the detection speed and reduces the resource requirements through model simplification and computational efficiency optimization. Checola et al. (2024) Focus on addressing the threat of Flavescence due to grapevine health and overcoming traditional monitoring methods’ high cost and low-efficiency issues. Develop an automatic pest detection system based on computer vision and deep learning.

Existing research on forestry pest detection mainly focuses on small-scale and fewer pest datasets. In contrast, research on large-scale and multi-class forestry pest detection is insufficient, which is insufficient to recognize and respond to multiple pest targets in real forestry scenarios. This study selected a large-scale forestry pest dataset as the training base. Twenty-nine different pest classes were covered. The dataset covers different stages of adults, larvae, and eggs of various pests. It brings significant challenges for model training and evaluation. To cope with these challenges, this paper proposes a forestry pest detection model based on improved YOLOv8n, which is designed to effectively deal with large-scale and multicategory forestry pest detection problems by deep learning techniques to achieve high-precision recognition of pests in complex and diverse forestry environments.

## 3 Materials and methods

### 3.1 Introduction to the dataset

**Figure 1:**
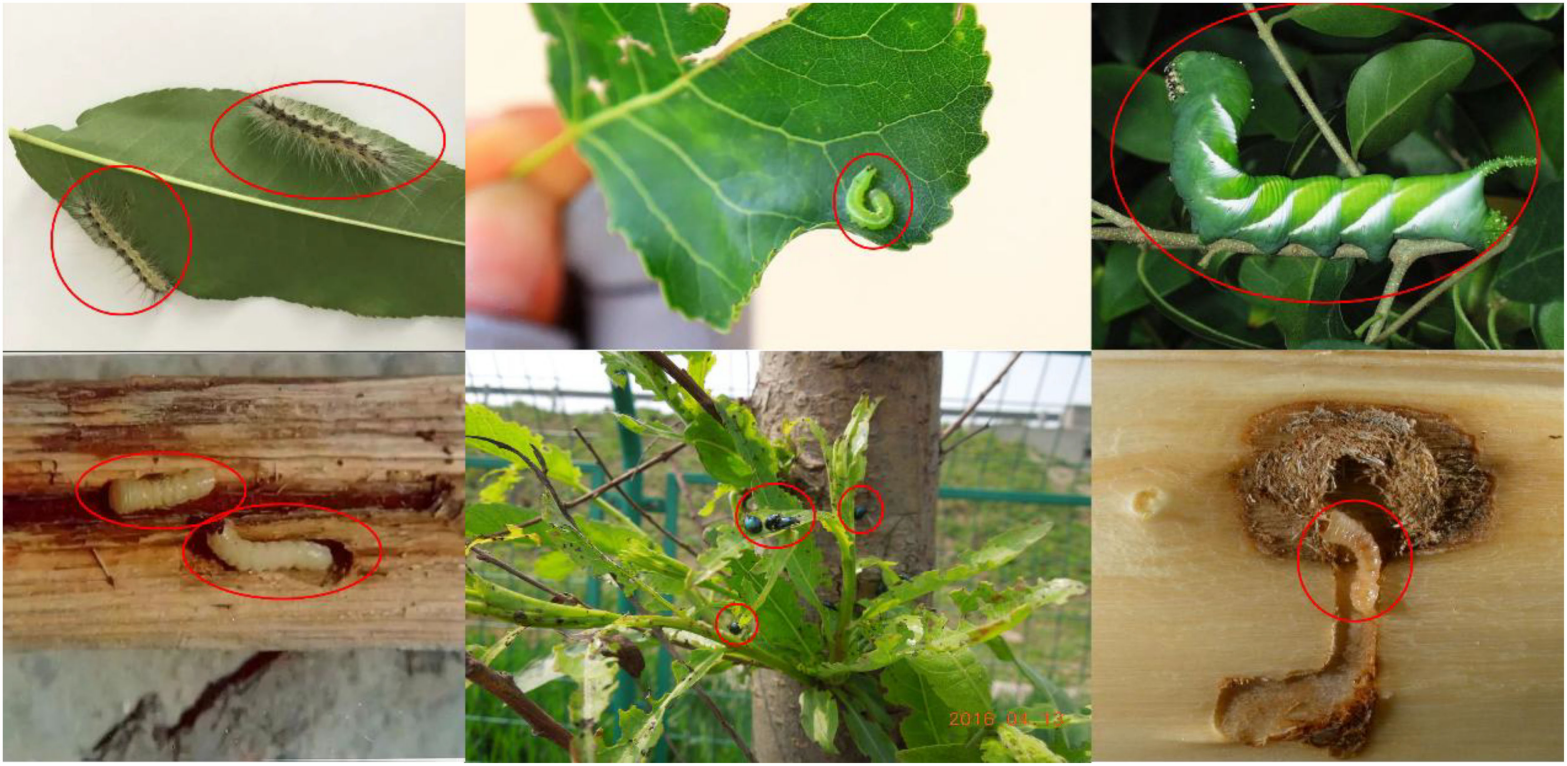
Image samples in the dataset.

The comprehensive dataset of multiple forestry pests proposed by (Liu et al., 2022) covers morphological characteristics of different life cycle stages with high-similarity backgrounds, shading, and dense targets. Data enhancement of the dataset involves applying various techniques to increase the diversity and quantity of data, thereby improving the model’s generalization ability. These techniques include geometric transformations such as rotation, flipping, translation, and scaling and image processing methods such as adding noise, adjusting brightness, contrast, and color dithering. To ensure the effectiveness and generalization of the model in practical scenarios, we further discussed the diversity of environmental conditions in the dataset. Although this dataset covers various forestry pests, in practical applications, pests may appear under different ecological conditions, such as other geographical locations, climate conditions, and tree species. Therefore, we analyzed the diversity of environmental conditions in the dataset, which includes different types of backgrounds, such as trees, leaves, and soil types, to simulate the complex environment of the real world. Some of the images of the dataset are shown in **Figure 1**. The dataset is randomly divided into training, testing, and validation sets in the ratio of 7:2:1. The specific division is shown in **Table 2**.

**Table 2:** Breakdown of training, testing, and validation set divisions.

Name	Train Tags	Val Tags	Test Tags
Drosicha_contrahens_female	417	67	128
Drosicha_contrahens_male	153	22	51
Chalcophora_japonica	110	16	34
Anoplophora_chinensis	316	46	86
Psacothea_hilaris (Pascoe)	174	20	37
Apriona_germari (Hope)	253	35	72
Monochamus_alternatus	138	23	39
Plagiodera_versicolora (Laicharting)	347	43	64
Latoia_consocia_Walker	205	41	44
Hyphantria_cunea	306	45	94
Cnidocampa_flavescens(Walker_pupa)	205	17	54
Erthesina_fullo	211	37	64
Erthesina_fullo_nymph-2	1749	309	482
Erthesina_fullo_nymph	142	17	59
Psilogramma_menephron	163	26	43
Sericinus_montela	269	51	76
Sericinus_montela_larvae	227	36	77
Clostera_anachoreta	223	23	62
Micromelalopha_troglodyta (Graeser)	174	24	40
Latoia_consocia_Walker_larvae	426	15	167
Plagiodera_versicolora(Laicharting)_larvae	618	73	187
Plagiodera_versicolora(Laicharting)_ovum	1963	380	637
Spilarctia_subcarnea(Walker)_larvae	123	31	36
Spilarctia_subcarnea(Walker)_larvae-2	320	16	86
Psilogramma_menephron_larvae	141	21	46
Cerambycidae_larvae	318	13	85
Micromelalopha_troglodyta(Graeser)_larvae	293	24	81
Hyphantria_cunea_larvae	240	46	154
Hyphantria_cunea_pupa	315	34	65

### 3.2 Assessment criteria

To better show precisely the advantages of the pruning algorithm and model improvement, the following performance evaluation metrics were used: precision (P), recall (R), calculation, parameters, and AP (Average Precision) averaged over mAP@.5:.95.

Precision is the ratio of correctly recognized forestry pest targets to all detected forestry pest targets by the network model, as shown in Equation 1. The recall represents the ratio of correctly recognized forestry pest targets to all labelled forestry pest targets, as shown in Equation 2. When delving into the performance evaluation of pest detection algorithms, special attention should be paid to three core indicators: TP (true positive), FP (false positive), and FN (false negative). Here is a detailed explanation of these indicators: TP (True Positive) indicator, which refers to the number of targets correctly identified and labelled as pests in the algorithm’s prediction results. In other words, TP reflects the algorithm’s accuracy in identifying actual pests. A high TP value means that the algorithm has high detection capability and can effectively identify pests, which is crucial for developing and implementing pest management strategies. The FP (false positive) indicator represents the situation where the algorithm incorrectly identifies nonpest objects as pests during the prediction process. In other words, FP refers to the number of samples that are not actually pests but are incorrectly labelled as pests by the algorithm. The higher the FP value, the higher the false alarm rate of the algorithm, which may lead to unnecessary prevention measures, increase costs, and potentially hurt the environment. The FN (false negative) indicator measures the actual number of pest targets that the algorithm fails to recognize during the detection process. That is to say, FN refers to the number of samples that are pests but have not been detected by the algorithm. The higher the FN value, the higher the missed detection rate of the algorithm, which may lead to the neglect of pest problems and affect the overall pest control effectiveness. The three indicators of TP, FP, and FN together constitute a comprehensive system for evaluating the performance of pest detection algorithms. By accurately calculating and interpreting these indicators, we can have a more comprehensive understanding of the advantages and disadvantages of the algorithm and then optimize it to improve the accuracy and efficiency of pest detection.


(1) $$Precision\;=\;\frac{TP}{TP+FP}$$

(2) $$Recall\;=\;\frac{TP}{TP+FN}$$

By considering the P and R metrics, AP evaluates the model’s performance in each category with a value equal to the area between the P-R curve and the axes. As shown in Equation 3.


(3) $$AP\;=\;1\int_{0}^{1}Precision\;\left(Recall\right)\;d\;\left(Recall\right)$$

Map@0.5:0.95(%) is the average of the APs calculated under mAP 0.5-0.95, which is the average of the APs of the IoUs (0.5-0.95 in steps of 0.05). In this experiment, F denotes the target category, and Map@0.5:0.95(%) is the harsher metric used to compare the detection of targets. The calculation formula is shown in Equation 4.


(4) $$mAP=\frac{1}{F}\sum_{i=1}^{F}AP_{i}$$

When evaluating neural network models built based on deep learning frameworks, in addition to considering accuracy metrics, the complexity of the model must also be considered. The number of network parameters (Parameters) is often used to describe the complexity of the model. “Lightweight” usually refers to reducing resources while maintaining functional integrity, reducing the resource footprint, or reducing the size and complexity of the system. FLOPs represent the speed of neural network models to perform computations, and the computational power, parameter decrease, and FLOPs decrease can be considered as an indication of lightweight. The specific objective of this study is to validate the lightweight performance of the improved YOLOv8.

### 3.3 YOLOv8 and its improved models

#### 3.3.1 YOLOv8 modeling

The Ultralytics team launched the YOLOv8 model in January 2023 with a structure that includes an input, backbone, neck network, and head module. The YOLO family balances speed and accuracy as a single-stage detection algorithm, allowing it to excel in target detection. The backbone of YOLOv8 utilizes the Darknet53 structure and contains the C2f, Conv, and SPPF modules. The C2f module, derived from the ELAN structure of YOLOv7, replaces the C3 module of YOLOv5 (Duan et al., 2023), providing rich gradient flow information and enhancing the feature representation capability. The SPPF module captures multi-scale context information through pyramid pooling operations to reduce information loss and computational burden. The neck module adopts the YOLOv5-like PAN and FPN design, eliminates the CBS 1x1 sampled on the PAN-FP (Lin et al., 2017; Liu et al., 2018), and replaces the C3 module with the C2f module to enhance the multi-scale information management and feature fusion capability. The detector head (DETECT) separates classification from detection through an anchorless design (Chen et al., 2023b), which directly predicts the aspect ratio and centroid of the target, improving the detection speed. The introduced distributed focusing loss reduces the sensitivity to outliers and improves the robustness of the model.

#### 3.3.2 RSD-YOLOv8

**Figure 2:**
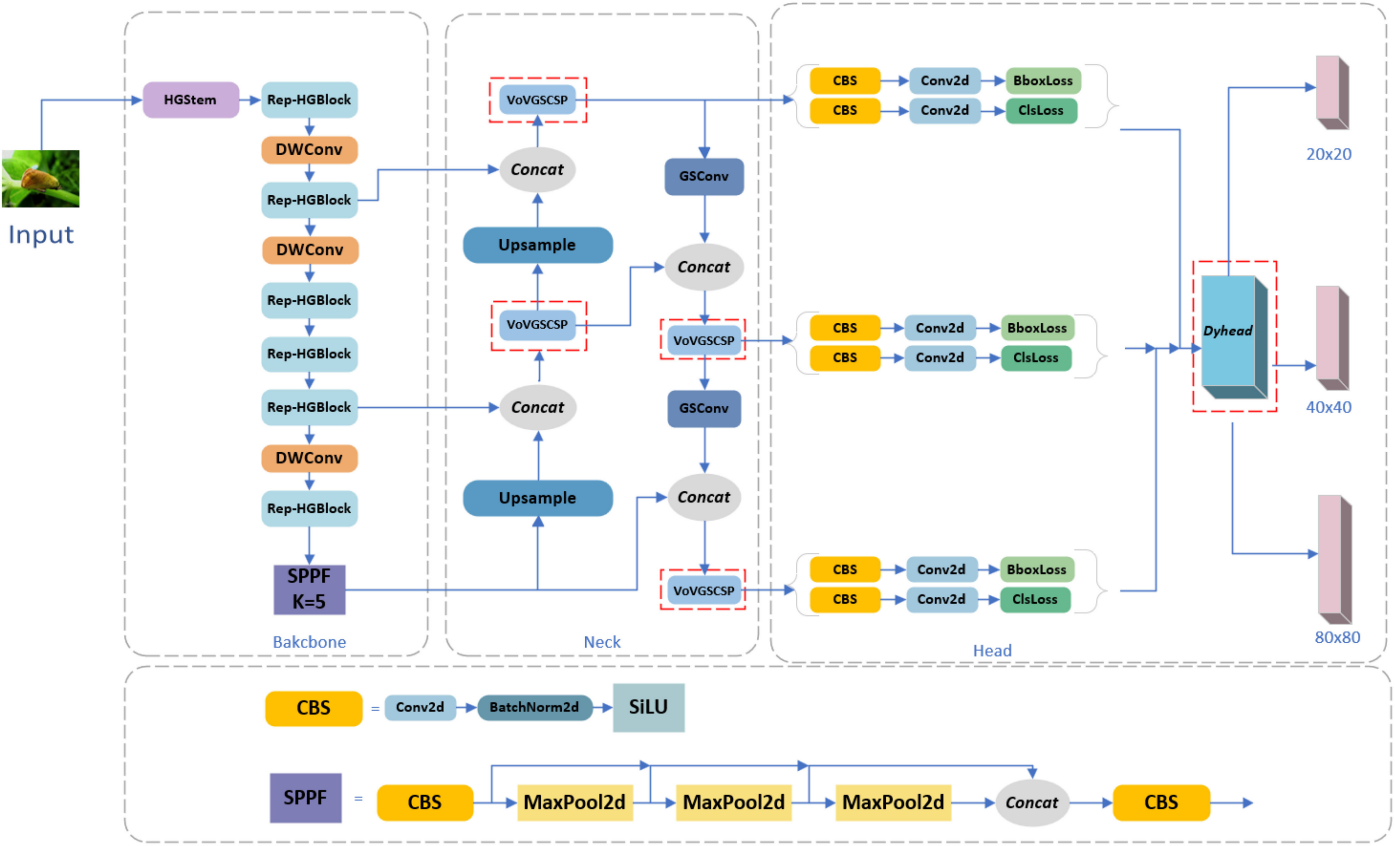
Improved YOLOv8 model (RSD-YOLOv8).

The HG block in HGNetV2 (Zhao et al., 2024) within RT-DETR is combined with RepConv to create Rep_HGBlock, significantly reducing the model parameters, computation, and size without sacrificing the detection accuracy. The Slim-neck module replaces the C2f and Conv modules in the original yolov8, which reduces the computational complexity in the forward propagation process The Slim-neck module replaces the C2f and Conv modules in the original yolov8, which reduces the computational complexity during the forward propagation process. And the detection accuracy of small targets is maintained or improved. Attention-based Dyhead is integrated into the detection head section to inject relevant attention on three perceptual dimensions: scale, space, and task. This integration of different self-attention mechanisms significantly enhances the feature extraction capability of the detection head. The model improvements are shown in **Figure 2**, and these enhancements enable the model to skillfully cope with the challenges in forestry pest detection scenarios and significantly improve the real-time detection efficiency. The model achieves high detection accuracy through algorithmic optimization while reducing the number of parameters, computational complexity, and model size. As a result, RSD-YOLOv8 is more suitable for meeting the high-precision pest detection needs of agricultural production, providing an efficient solution.

### 3.4 Model improvements

#### 3.4.1 Rep-HGNetV2

**Figure 3:**
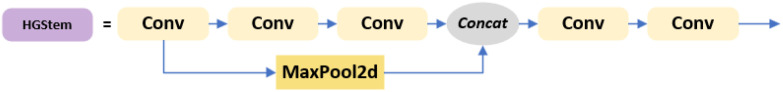
HGStem module structure.

Improving model efficiency and performance remains a key research area in deep learning and computational vision. Therefore, a novel backbone network Rep-HGNetV2 consisting of HGStem, Rep-HGblock, and DWConv was designed. As shown in **Figure 3**, the HGStem module includes five key convolutional operations and a max pooling operation, which helps to extract low-level features efficiently and effectively. The convolution and pooling operations efficiently extract the basic image features. The HGStem module provides a robust approach for feature extraction in complex neural network architectures, enabling efficient input data encoding and improving the computational efficiency of the entire network without compromising performance.

**Figure 4:**
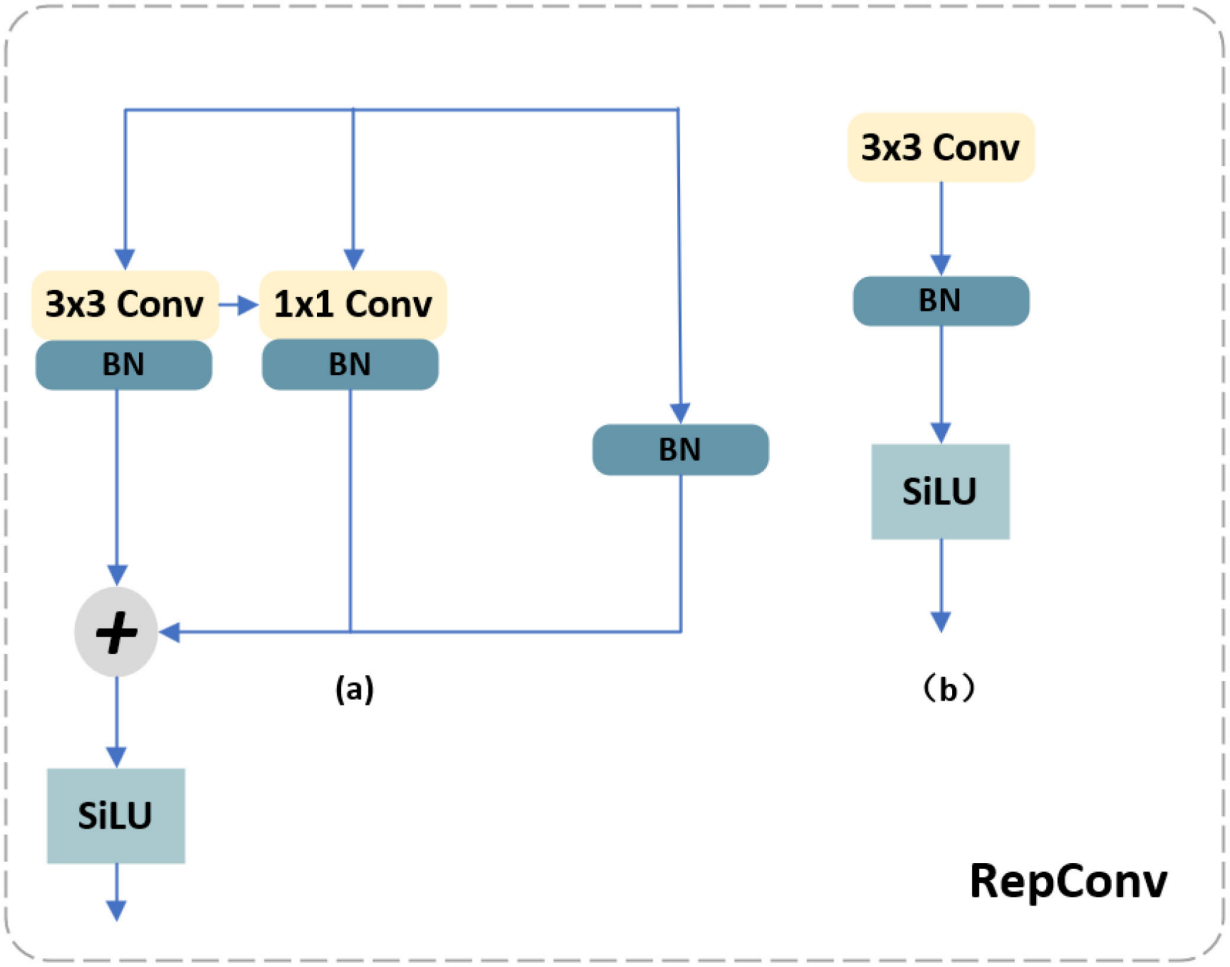
RepConv module structure **(a)** Training phase; **(b)** Inference Phase.

The core idea of RepConv comes from RepVGG, proposed by (Ding et al., 2021), a technique specifically designed for model reparameterization. It can transform a complex convolutional neural network into a network consisting of simple convolutional and fully connected layers. This reparameterization process significantly reduces the computational effort and the number of parameters in the model, resulting in improved inference speed and lightweight deployment capabilities. The core concept is to replace the original convolution operation with a module consisting of a convolutional layer and an element-wise addition operation to realize the reparameterization of the network. This reduces computation and memory consumption. As shown in **Figure 4**. the RepConv module integrates RepVGG into the feature fusion network. During the training process, several branches are used, including a 3x3 convolutional kernel module, a 1x1 convolutional kernel module, and shortcuts. The BN layer follows the convolutional layer. The first step of reparameterization is to convert 1x1 convolution and shortcuts to 3x3 convolution kernels that output the same result. In the inference phase, structural reparameterization merges each RepConv block into a 3x3 CBS module. The multi-branch topology allows learning rich feature information during training, while the simplified single-branch architecture reduces memory consumption and enables fast inference during the inference phase.

**Figure 5:**
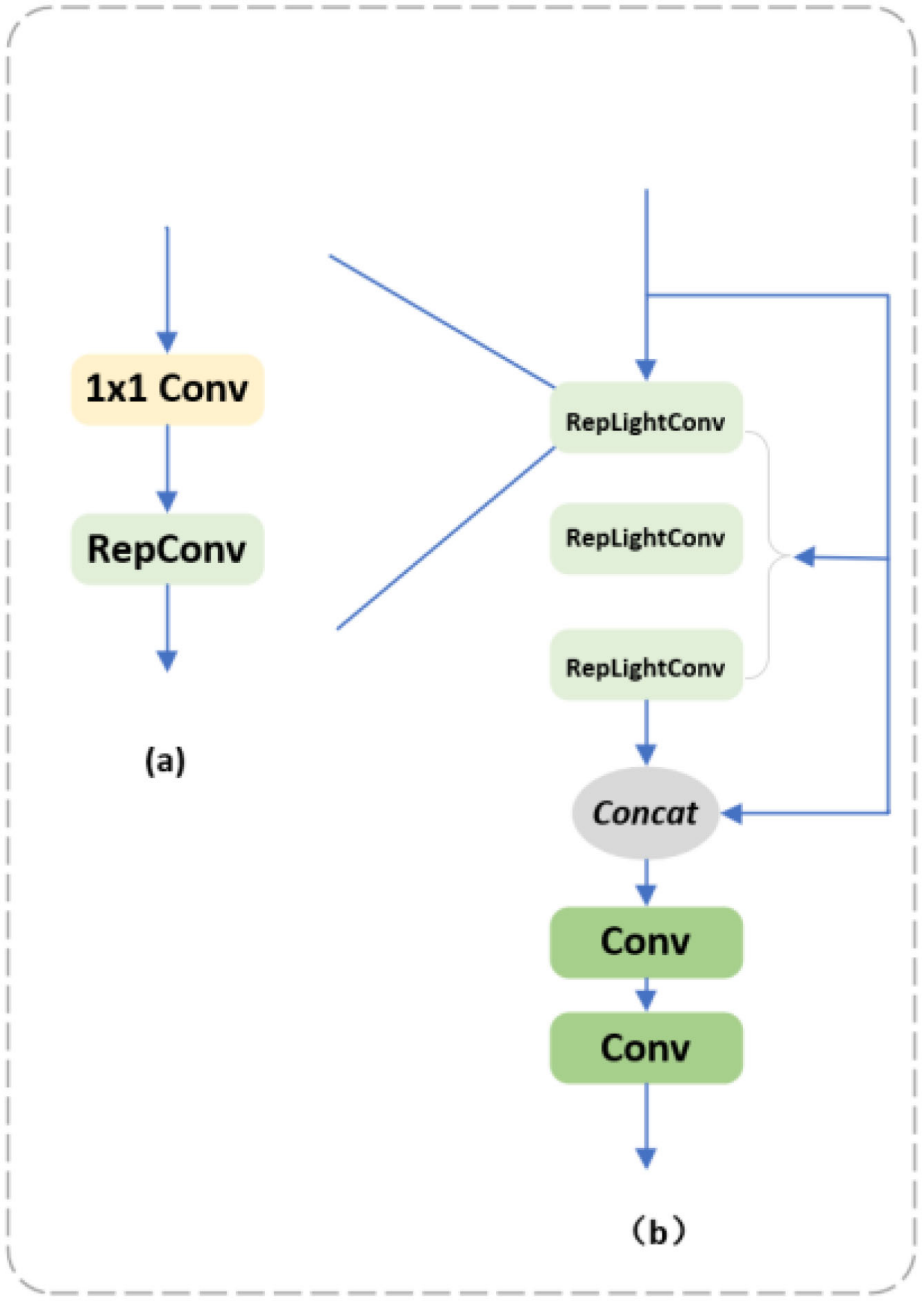
**(a)** RepLightConv; **(b)** Rep-HGBlock.

The traditional convolution has the disadvantages of many parameters, high computation, and high memory consumption, as shown in **Figure 5a**. The proposed RepLightConv maintains a high model representation capability while reducing the parameters and computation by combining 1x1 convolution and RepConv. 1x1 convolution efficiently reduces the number of parameters between the channels, while RepConv further optimizes the computational efficiency. The number of parameters is significantly reduced while maintaining high performance. As shown in **Figure 5b**, Rep-HGblock replaces the regular convolution in the original HGblock module in HGNetV2 with the reparameterized RepLightConv convolution, which significantly reduces the number of parameters in the model and lowers the memory occupation compared with the traditional convolution module; secondly, it improves the computational efficiency, making The model is more efficient in the training and inference process; despite the reduced number of parameters, RepLightConv still maintains a high model expressiveness for resource-constrained environments, such as mobile devices or embedded systems. With these improvements, Rep_HGBlock significantly reduces computation and memory usage while maintaining high performance, allowing it to excel in resource-constrained scenarios.

In this study, several mainstream backbone networks are tested under the YOLOv8 framework to evaluate the full performance of RepHGNetV2. These backbone networks include YOLOv8’s original CSPDarkNet53 (Shen et al., 2023), the lightweight MobileNetV3 and MobileNetV4, and Vanillanet and StarNet. Although MobileNetV3 stands out for its lowest parameter count (1.19M), computational complexity (2.4G), and model size (2.6MB), its performance in key experimental metrics such as precision (90.2%), recall (53.2%) and Map@0.5:0.95 (69.8%), etc., is not satisfactory. In contrast, although Vanillanet and StarNet have advantages over RepHGNetV2 in terms of the number of parameters, computational complexity, and model size, the experimental results show a significant performance gap between them and RepHGNetV2. After comprehensive comparison and analysis, RepHGNetV2 achieves the best balance between accuracy, computational complexity, parameter complexity, and model size. Therefore, it was finally decided to replace the original CSPDarkNet53 backbone with RepHGNetV2 in YOLOv8 to achieve better overall performance. The experimental results are shown in **Table 3**.

**Table 3:** Comparison of different backbone networks.

References	Model	P (%)	R(%)	Map@0.5:0.95(%)	Params(M)	FLOPs (G)	Weight Size(MB)
Howard et al. (2019)	MobileNetV3	90.2	83.2	69.8	1.19	2.4	2.6
Shen et al. (2023)	CSPDarkNet53	97.0	94.8	84.4	3.01	8.1	6.0
Chen et al. (2023a)	Vanillanet	93.3	86.4	70.3	1.73	5.1	3.5
Ma et al. (2024)	StarNet	95.6	93.6	82.2	2.21	6.5	4.7
Qin et al. (2025)	MobileNetV4	95.5	92.5	81.9	5.70	22.6	11.8
/	RepHGNetV2	97.8	95.2	85.3	2.34	6.9	4.8

#### 3.4.2 Slim-neck module

Forestry pest detection demands rapid recognition speeds and high model accuracy to facilitate prompt preventive measures. For future applications involving real-time monitoring of forestry pests on embedded devices, it is crucial to maintain model performance while achieving smaller model sizes and faster algorithm execution. Li et al. (2022b) proposed the GSConv approach to address model complexity, leveraging the synergistic use of standard and depthwise separable convolution. Standard Convolution (SC) is known for its fusion and feature extraction capabilities, yet its overuse leads to a substantial accumulation of parameters. Conversely, Depthwise Separable Convolution (DSC) significantly reduces the model’s overall parameters and floating-point operations, albeit with a trade-off in channel information loss. The GSConv approach ingeniously combines SC and DSC with a shuffle operation to form a unified module. This combination effectively mitigates the complexity of the model, ensuring a balanced reduction in parameter count and computational load while preserving essential channel information. Such advancements are pivotal for deploying efficient, high-performance models on resource-constrained embedded devices, enhancing forestry pests’ real-time detection and monitoring.

The following Equations 5–7 show the operations’ SC, DSC, and GSConv complexity.


(5) $$Time_{SC}∼O\left(W\times H\times K_{1}\times K_{2}\times C_{1}\times C_{2}\right)$$

(6) $$Time_{DSC}∼O\left(W\times H\times K_{1}\times K_{2}\times 1\times C_{2}\right)$$

(7) $$Time_{GSConv}∼O\left[W\times H\times K_{1}\times K_{2}\times \frac{C_{2}}{2}\left(C_{1}+1\right)\right]$$

In the context of convolutional operations, let W and H represent the width and height of the output feature map, respectively. The convolutional kernel size is denoted by $K_{1}\times K_{2}$ . The term $C_{1}\times C_{2}$ refers to the number of channels of the input feature map, which also corresponds to the number of channels associated with each convolution kernel. Meanwhile, $C_{2}$ indicates the number of channels of the output feature map. In a standard convolution (SC) context, generating each output feature map channel requires convolution across all input channels, and then the results are accumulated. This approach results in substantial computational overhead, mainly when the number of input and output channels $C_{1}\;$ and $C_{2}$ respectively is large. In contrast, GSConv mitigates this computational burden by halving the number of output channels( $\frac{C_{2}}{2}$ )and optimizing the input and output channel relationship $\;C_{1}+1$ , thereby significantly reducing computational complexity compared to SC.

Depthwise Separable Convolution (DSC) addresses computational inefficiencies by decomposing the convolution operation into depth and pointwise convolution. This decomposition dramatically reduces the parameter count and computational complexity, especially for larger values of $\;C_{1\;}$ and $\;C_{2}$ . On the other hand, GSConv further reduces computational complexity by finetuning the relationship between $\;C_{1\;}$ and $\;C_{2\;}$ while maintaining performance. This approach results in further compression of computational complexity without compromising model accuracy.

**Figure 6:**
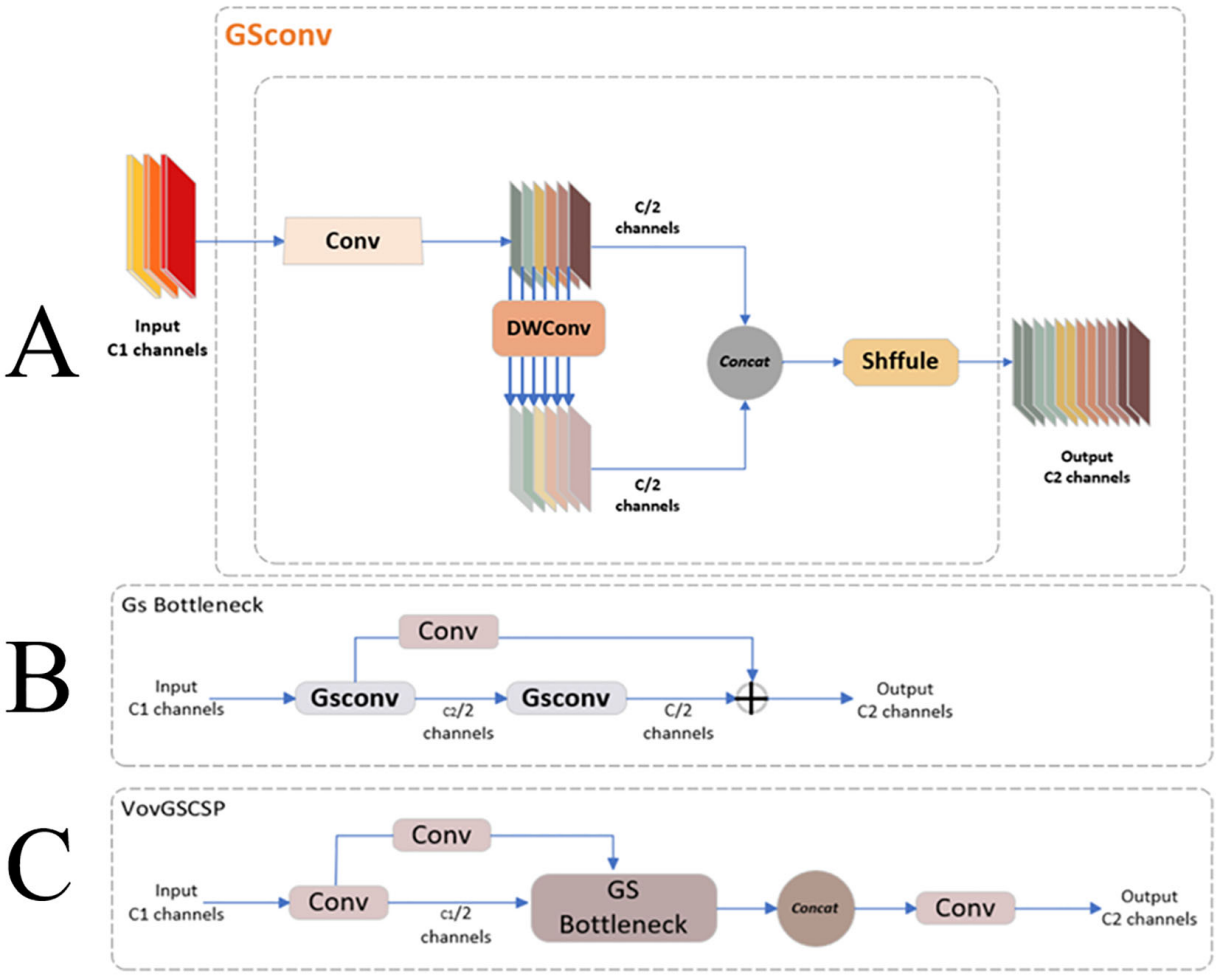
**(A)** GSConv model structure **(B)** GSbottleneck model structure **(C)** VoVGSCSP model structure.

GSConv demonstrates superiority over traditional SC and DSC in specific applications by introducing additional computational optimizations and structured sparsity, building upon the foundational principles of deep separable convolution. These enhancements lead to higher computational efficiency and potential performance gains, underscoring the advantages of GSConv in reducing computational demands while preserving or enhancing overall performance. GSConv effectively balances model accuracy and computational speed, as illustrated in **Figure 6A**. This architecture enables the model to remain lightweight while preserving accuracy. The mechanism involves fusing the output information from Standard Convolution (SC) with that of Depthwise Separable Convolution (DSC) through a shuffle operation. The shuffle operation acts as a homogeneous mixing strategy, ensuring that the information derived from SC is seamlessly integrated into the DSC output. This process facilitates the uniform exchange of local feature information across different channels, optimizing the network’s overall performance.

To further enhance inference speed while preserving accuracy, the GSConv framework incorporates the GSbottleneck module, which forms the core of the VoVGSCSP module. This module comprises the GSbottleneck, CONV, and Concat modules, as depicted in **Figure 6B**. The GSbottleneck integrates two GSConv layers with added skip connections to streamline the computational process. Specifically, the first GSConv layer reduces the number of input channels by half, and subsequently, the second GSConv layer processes these reduced channels. This design ensures efficient channel reduction and processing, contributing to faster inference times while maintaining the model’s accuracy. In **Figure 6C**, the VoVGSCSP module’s structure, which leverages the GSbottleneck’s efficient channel handling, the CONV module’s convolutional capabilities, and the Concat module’s feature aggregation, exemplifies an optimal balance between speed and accuracy in the GSConv architecture.

In the VoVGSCSP module, the input feature map is divided into two segments, each comprising a subset of the total channels. The first segment undergoes a convolution operation and feature extraction through stacked GSbottleneck modules. Concurrently, the second segment is a residual connection, passing through a single convolution layer. These two segments are subsequently fused and concatenated channel-wise, and the combined feature map is output through an additional convolution layer. This design results in a more robust nonlinear representation compared to the c2f module, effectively addressing the issue of vanishing gradients. The VoVGSCSP module reduces the number of parameters and maintains measurement accuracy (Zhang et al., 2023b), which is crucial for deploying models on embedded devices. This makes it optimal for applications requiring efficient computation and high performance.

#### 3.4.3 Dynamic head module

For different stages of forestry pests, the size of the detection target varies, and Dynamic Head (Dai et al., 2021) solves this problem by using level-wise detection. The characteristic of level-wise sensing is that different feature maps correspond to different scale sizes, and improving the detector’s level-wise sensing ability also adjusts the scale expression ability at the same time. As shown in Equation 8.


(8) $$\pi_{L}\left(F\right)\cdot F=\sigma \left(f\left(\frac{1}{SC}\sum_{S,C}F\right)\right)\cdot F$$

Where f(·) is a linear function composed of approximate 1*1 convolutions and σ(x)=max(0,min(1,(x+1)/2)) is the hard sigmoid function


(9) $$\pi_{S}\left(F\right)\cdot F=\frac{1}{L}\sum_{l=1}^{L}\sum_{k=1}^{K}w_{l,k}\cdot F\left(l;p_{k}+\Delta p_{k};c\right)\cdot \Delta m_{k}$$

Pest detection targets may appear at different positions in the detected image. The spatial wise feature in Dyhead better handles the spatial differences between different pests in the pest image features. Based on the feature maps corresponding to different positions, improving the spatial position perception ability of the detection head is achieved by changing the expression ability of different spaces. As shown in Equation 9. K is the number of sparsely sampled locations for which a $p_{k}\text{Δ}p_{k}\;$ position shift was done to focus on discriminative regions and $m_{k}$ is a self-learning importance metric on the location $p_{k}$ of self-learnable importance metric, they were appealing that both parameters can be F intermediate level of input feature learning.

The detection task contains different task information features, and the task aware (channel-wise) in Dyhead uses different detection heads to match them, using different feature channels for various classes of pests. As shown in Equation 10.


(10) $$\begin{array}{c} \pi_{C}\left(F\right)\cdot F=max(\alpha^{1}\left(F\right)\cdot F_{c}+\beta^{1}\left(F\right),\alpha^{2}\left(F\right)\cdot F_{c}\\ +\beta^{2}\left(F\right)) \end{array}$$

${\left[\alpha^{1},\alpha^{2},\beta^{1},\beta^{2}\right]}^{T}=\theta \left(\cdot \right)$ is a hyperfunction that learns to control the activation threshold, which is the role of the training process in learning how to control the threshold. θ(·) The specific role is to perform global pooling in L x S dimensions to reduce the dimensionality. Sequentially, it passes through two fully connected layers and a normalization layer and finally normalizes the output to [-1,1] using an offset sigmoid.

**Figure 7:**
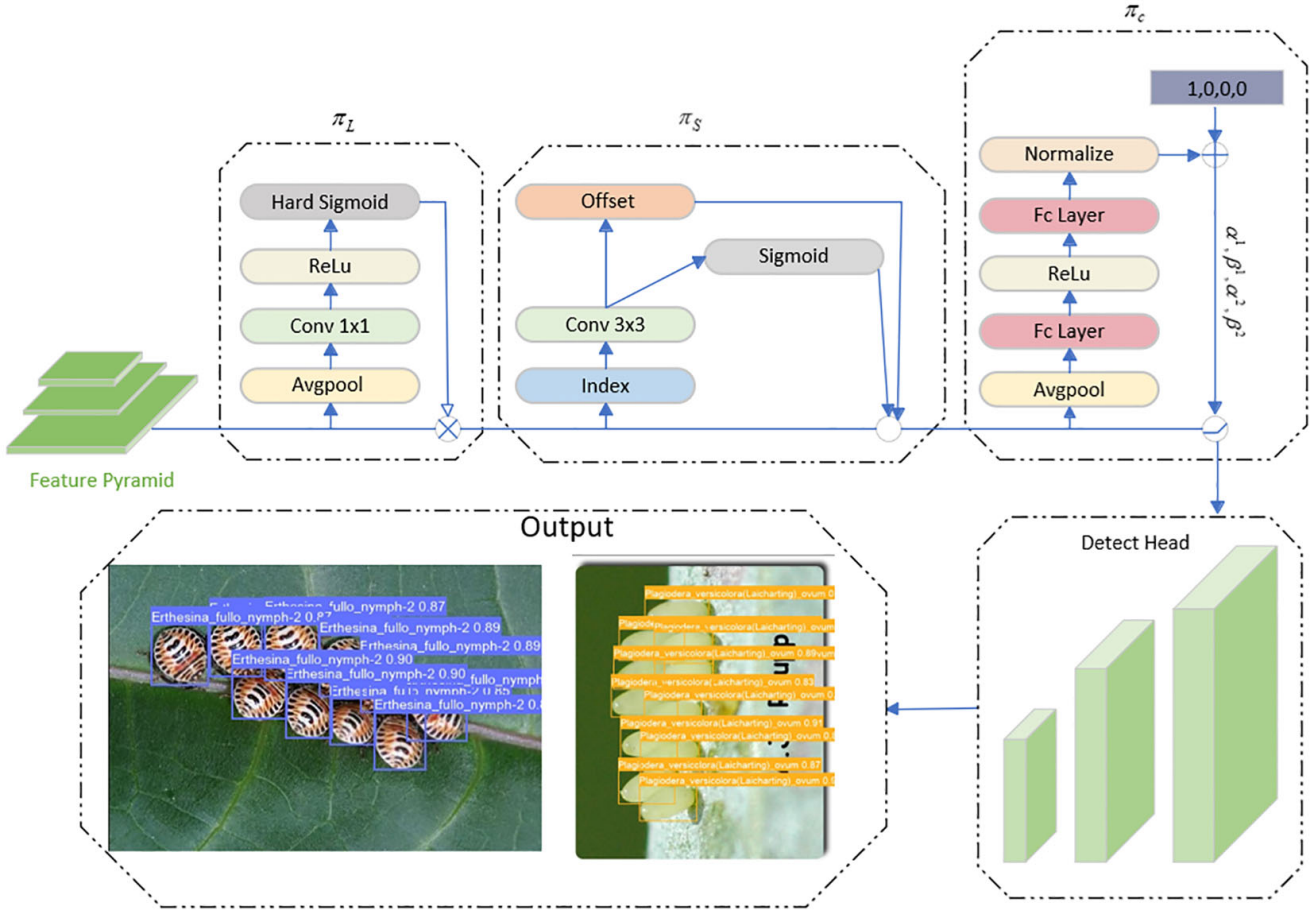
Dynamic head detection head workflow.

The Dynamic Head workflow is shown in **Figure 7**. $\;\pi_{L}$ and $\pi_{S}$ and $\pi_{C}$ corresponds to the level-wise, spatial-wise, and channel-wise attention modules.

As shown above, three perceptual modules augment the output by continuously stacking and combining the different attention locations to get the final result.

#### 3.4.4 Model pruning

In this experiment, a magnitude-based adaptive pruning algorithm (Lamp) proposed by (Lee et al., 2020) was used to prune the improved model. The method optimizes pruning efficiency by selecting the sparsity of the layers using the LAMP score. The technique can determine the sparsity of each layer by itself and has the advantages of high computational efficiency, no need to adjust hyperparameters, and no dependence on model-specific knowledge over traditional pruning methods.

Since the pruning method is layer-based adaptive magnitude pruning in the direction of structured pruning, the idea behind implementing LAMP is that a model-level distortion minimization perspective is adopted to treat the magnitude-based pruning. By expanding the weight tensor $W^{\left(i\right)}$ is expanded into a one-dimensional vector, which is subsequently ranked to satisfy  |W[u]|≤|W[v]| , which is the same as u <  v holds simultaneously, yielding a Lamp score that serves to exhibit the sparsity of each layer. The weight tensor W The Lamp score for the first index of the u LAMP score for an index is shown in Equation 11.


(11) $$\text{score}\left(u;W\right):=\frac{{\left(W\left[u\right]\right)}^{2}}{\sum_{v\geq u}{\left(W\left[v\right]\right)}^{2}}$$

**Figure 8:**
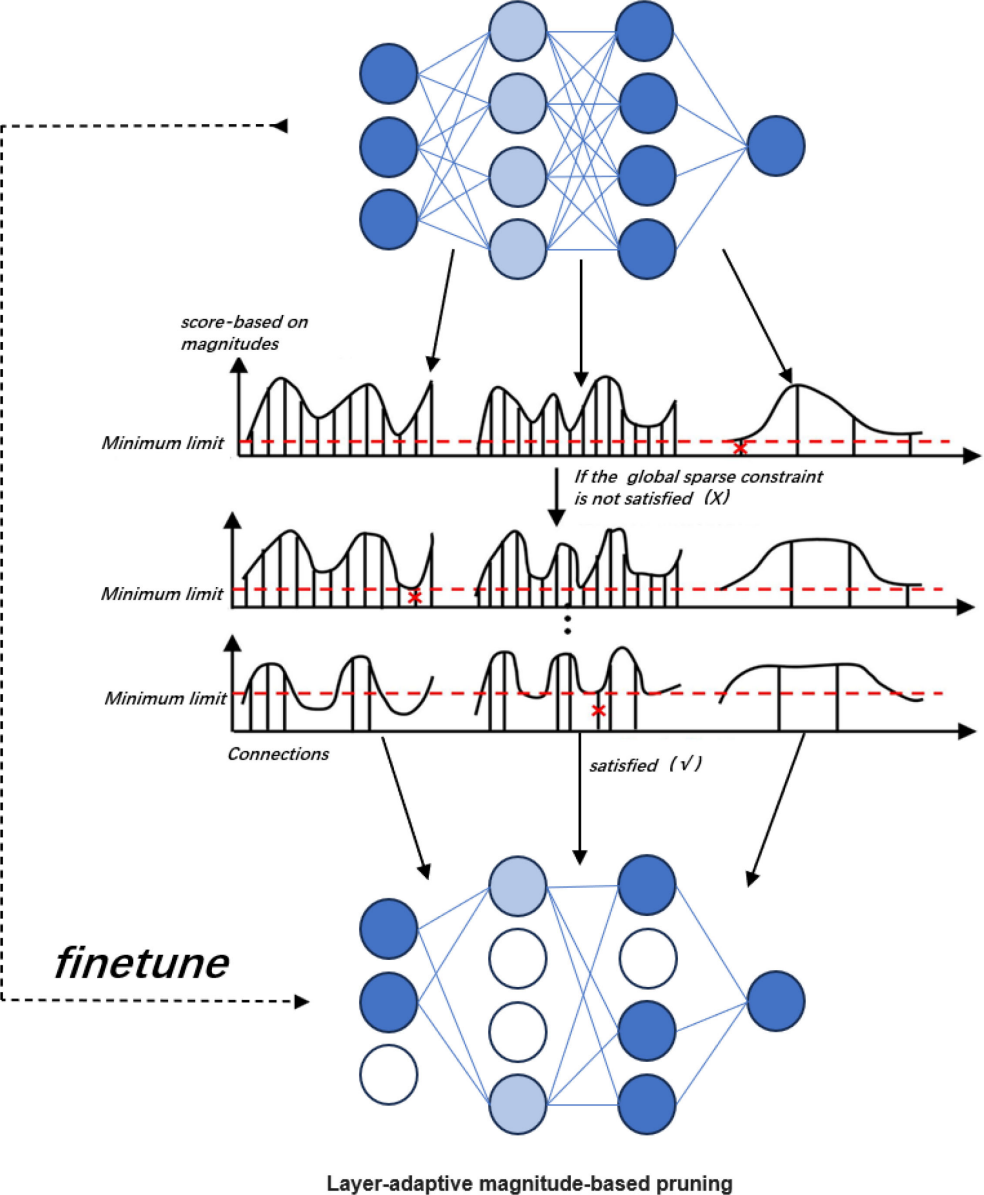
Schematic diagram of the LAMP pruning process.

The Lamp score is a rescaled weight magnitude metric, similar to the distortion of the pruned model caused by pruning. The Lamp score weighs the relatively meaningful target connections among all the residual connections belonging to the same layer, where connections with lower values of the weight magnitude are discarded. Two connections with the same weight magnitude have different Lamp scores. Once the Lamp scores are computed, they are sorted from the lowest to the highest scores, and according to the global sparsity constraints, all the LAMP score connections with less than the target weights are removed in order until the desired global sparsity constraints are satisfied, achieving a significant reduction in parameters. The pruning process is shown in **Figure 8**.

## 4 Experiments and analysis of results

### 4.1 Experiment and parameterization

All model training and testing procedures were executed on the same device with an experimental configuration of Inter 12700KF CPU, 32GB RAM, and NVIDIA GeForce RTX 3090TI GPU, and a training environment of Windows 11 (64bit) using Python 3.8, PyTorch 2.1.2 and CUDA 12.1.

During training, we normalized the input image size to 640×640. We used 16 batch sizes and trained the model for 300 epochs. The training-specific parameters are shown in **Table 4**.

**Table 4:** Training parameters.

Parameter	Value
Epochs	300
Batch size	16
Image size	640 x 640
Optimizer algorithm	SGD
Learning rate	0.01
Momentum	0.937
Weight decay	0.005

### 4.2 Ablation experiments

To better determine the impact of the improved module on the overall model, its effectiveness was assessed through ablation experiments (Huang et al., 2024). The results of the experiment are shown in **Table 5**.

**Table 5:** Comparative data of ablation experiments.

Model	P (%)	R (%)	Map@0.5:0.95 (%)	Parameters (M)	FLOPs (G)	Weight Size (MB)
YOLOv8n (baseline)	97.0	94.8	84.4	3.01	8.1	6.0
RepHGNetV2	97.8	95.2	85.3	2.34	6.9	4.8
slimneck	97.2	95.0	86.0	2.80	7.3	5.6
Dyhead	97.6	95.1	86.7	3.49	9.7	6.9
RepHGNetV2+slimneck	97.5	93.3	85.3	2.13	6.1	4.4
RepHGNetV2+Dyhead	98.0	94.4	86.6	2.82	8.5	5.7
slimneck+Dyhead	97.2	95.0	86.5	3.18	8.5	6.4
RSD-YOLOv8	97.6	94.6	86.0	2.61	7.6	5.4
RSD-YOLOv8 (1.5x)	98.0	95.6	88.6	1.91	5.2	4.0

It is clear from **Table 4** that the model, after replacing the RepHGNetV2 backbone network, drops by 21%, 15%, and 20% in parameters and operations and model size, respectively. The addition of the Dynamic Head module by Yolov8 yields a significant boost. mAP@.5:.95 improved by 2.3% in exchange for the boost at the cost of Params, GFLOPs, and FPS went up by 14%, 17%, and 13%. It is demonstrated that the inclusion of this module leads to an increase in computational cost and model size. A comparison of the experimental results for the slim-neck module, which is lightly optimized for the neck, shows that adding the slim-neck helps reduce the computational cost, with GFLOPs decreasing by 10% and the corresponding metrics decreasing differently compared to the Dynamic Head module.

**Figure 9:**
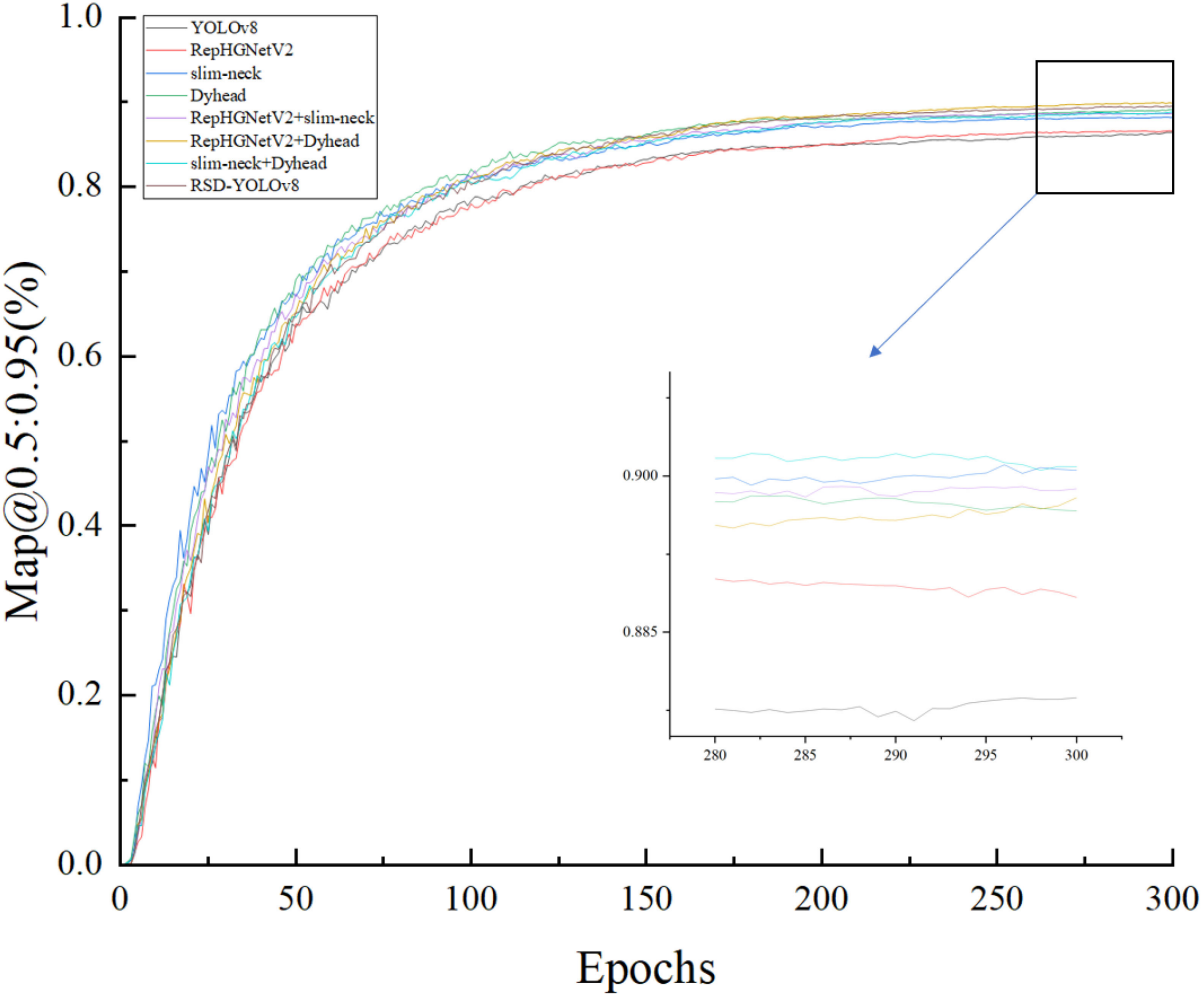
Training without pruning models Map@0.5:0.95 (%) values.

As shown in **Figure 9**, after about 300 epochs, Map@0.5:0.95(%) value reaches about 90% respectively and gradually stabilizes. It is far beyond the effect of using the module alone. It shows that the YOLOv8-Flight model has a high overall accuracy in forestry pest detection.

### 4.3 Pruning effect

After completing the ablation experiments, it was found that the parameters and operations of the model were too large, resulting in a model that was not lightweight enough, thus affecting its efficiency in practical applications (Zeng et al., 2023). The model pruning technique is introduced to reduce the complexity of the model by removing redundant and unimportant weights or neurons. The number of parameters and computations is decreased significantly. RSD-YOLOv8 was trained for 300 rounds to obtain the pre-trained weights. A pruning operation was performed on the weights obtained in training and then finetuned for 300 rounds (the same number of rounds as in training). Speed_up (FLOPS before pruning/FLOPS after pruning) was set to 1.5 times. The goal is to minimize the number of parameters and arguments without losing accuracy. While pruning technology improves model efficiency, it also brings some limitations. Firstly, pruning may unintentionally remove some weights or neurons that significantly impact model performance, resulting in information loss, which may hurt the model’s generalization ability. Secondly, balancing model size and improving accuracy is a highly challenging task. When Speed_up is set too high, a lightweight model can be obtained. Still, excessive pruning may lead to a significant decrease in model performance and fail to achieve the expected accuracy improvement.

The results of the pruning experiment are shown in **Table 6**. Model pruning is performed on the improved model base. The results obtained were compared with the baseline without loss of accuracy. Comparison of the baseline model (YOLOv8n) with the enhanced model RSD-YOLOv8 (1.5x) showed a reduction in parameters from 3.01 to 1.91 (37%), FLOPS from 8.1 to 5.2 (36%), weight size from 6.0 to 4.0 (33%), and Map@0.5:0.95(%) increased from 84.4% to 88.6% (4.2%). The above operation effectively simplifies the model size. The above operation effectively simplifies the model size and improves the computational efficiency without losing the detection accuracy. Significantly improves mAP@.5:.95, a demanding target detection evaluation metric. It can be proved that introducing these two modules and the pruning algorithm can achieve the best detection performance on this dataset, thus justifying the three improvements paired with the pruning algorithm.

**Table 6:** Comparative data from pruning experiments.

Model	P (%)	R (%)	Map@0.5:0.95 (%)	Parameters (M)	FLOPs (G)	Weight Size (MB)
RepHGNetV2 (1.5x)	98.3	95.3	86.0	1.55	4.6	3.3
slimneck (1.5x)	97.4	95.5	86.8	1.98	4.9	4.1
Dyhead (1.5x)	97.7	95.3	87.9	2.36	6.4	5.0
RepHGNetV2+slimneck(1.5x)	97.7	95.1	87.6	1.54	4.1	3.3
RepHGNetV2+Dyhead (1.5x)	98.4	94.8	87.9	1.91	5.5	4.0
slimneck +Dyhead (1.5x)	97.8	95.4	87.7	2.35	6.0	5.1
RSD-YOLOv8 (1.5x)	98.0	95.6	88.6	1.91	5.2	4.0
RSD-YOLOv8 (2.0x)	97.3	94.7	87.6	1.51	3.9	3.2

**Figure 10:**
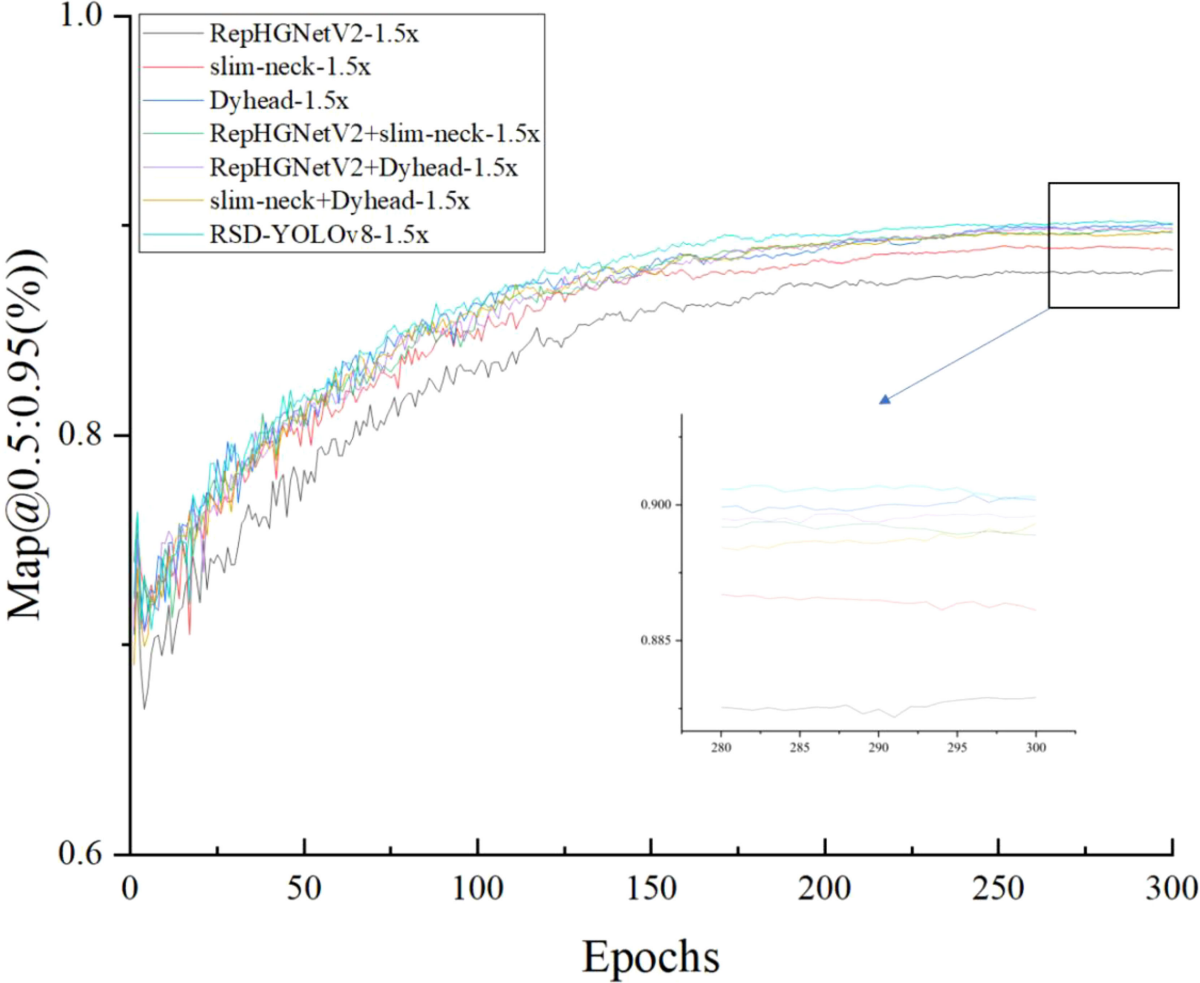
Training with 1,5x pruning models Map@0.5:0.95 (%) values.

**Figure 10** shows that Map@0.5:0.95(%) will not be 0 to start growing because the Lamp pruning algorithm will first process the model to reduce the precision, model size, and the number of operations, and at the end of the compressed model, finetune processing.

**Figure 11:**
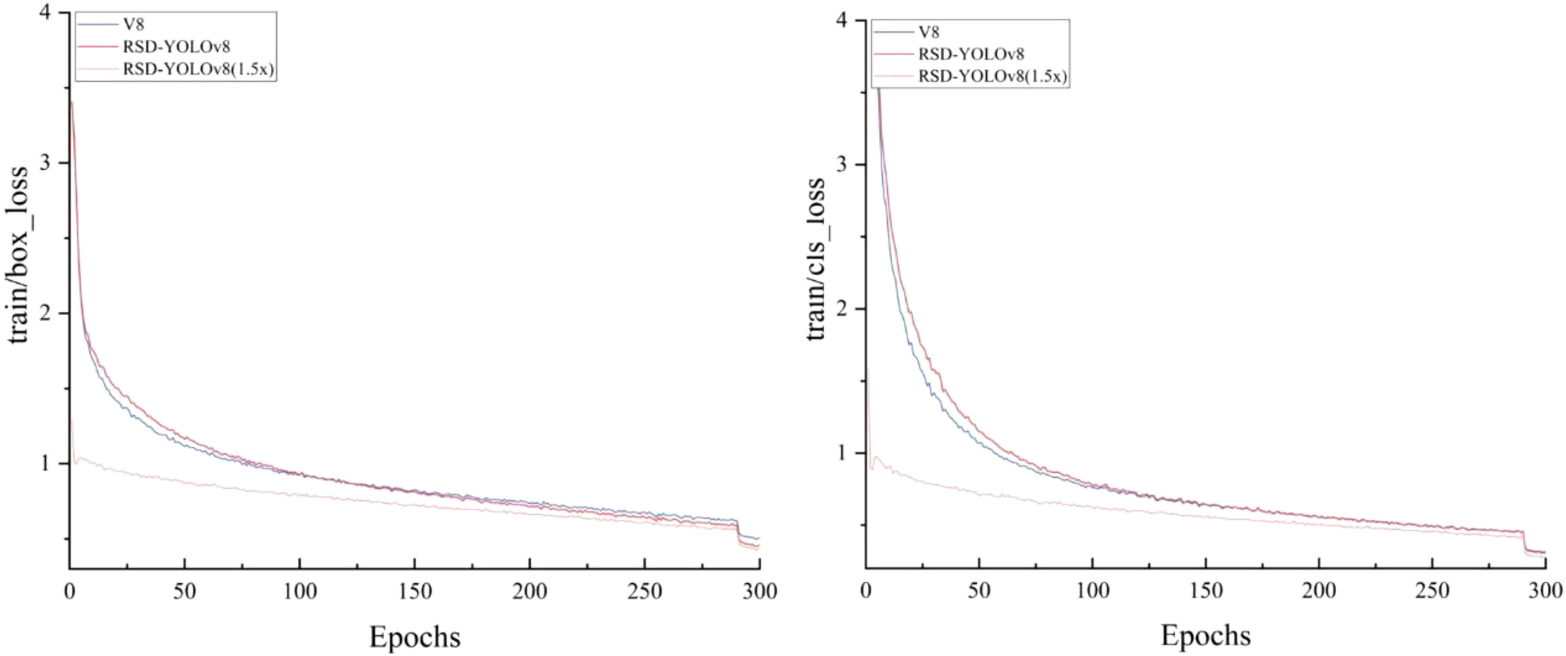
Training loss curve.

**Figure 11** shows the training loss curves (prune) for YOLOv8, RSD-YOLOv8 and RSD-YOLOv8(1.5x). The loss values tend to stabilize at 300 iteration cycles, indicating that the training has converged without overfitting. The decrease in the loss values for the last 10 rounds of the training set is due to the removal of the mosaic enhancement being turned off over the previous 10 rounds of training, which improves the stability of the model and reduces unwanted noise in the later stages of training.

**Figure 12:**
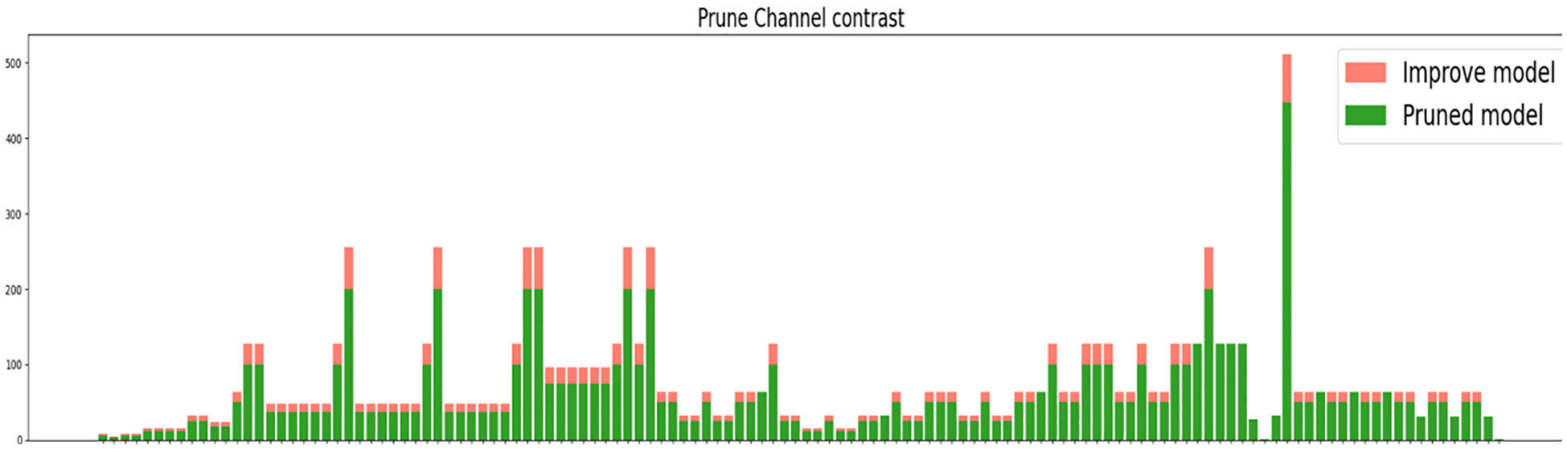
Comparison before and after.

In **Figure 12**, the horizontal coordinates indicate the name of each layer, and the vertical coordinates indicate the number of channels; the number of channels before trimming is shown in green, and the number of channels after trimming is shown in pink. After the trimming operation using Lamp (1.5x), it is possible to visually compare the channels in each layer; most are compressed differently.

**Figure 13:**
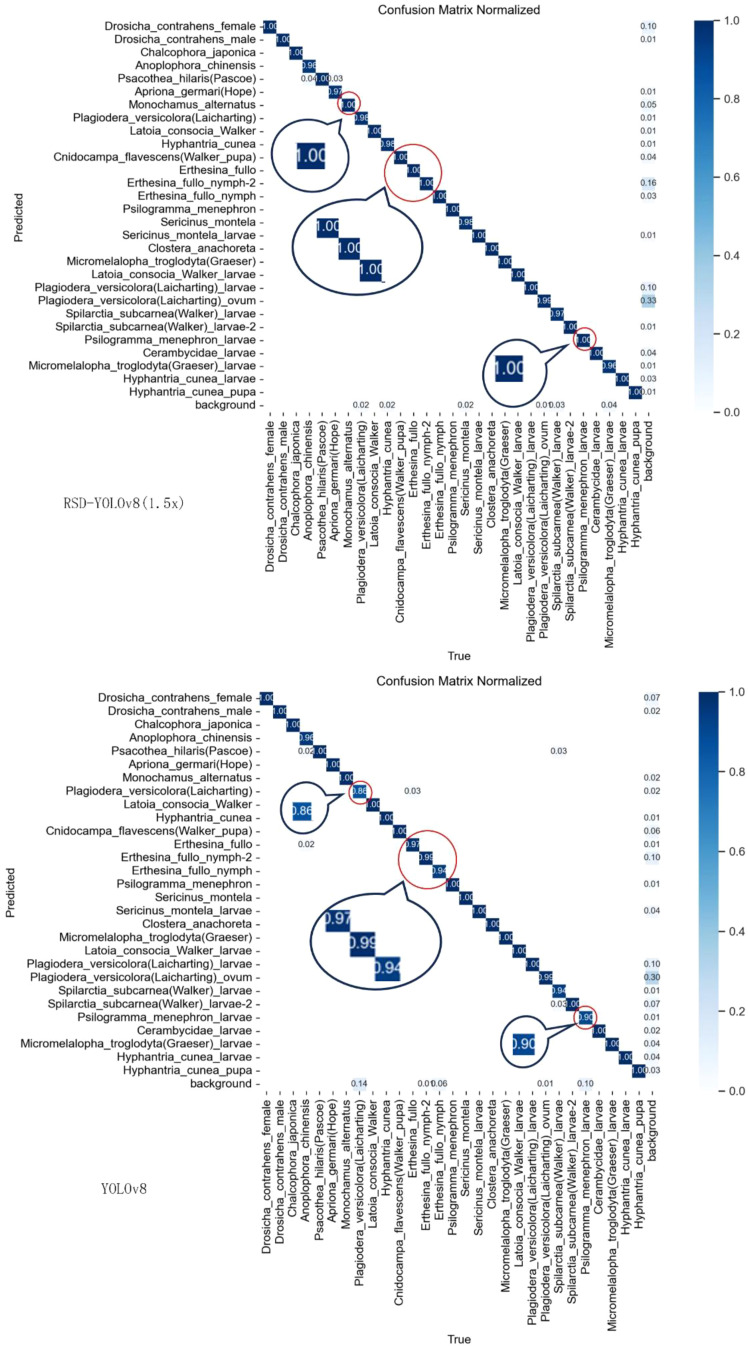
Comparison of confusion matrices.

The confusion matrix is the most intuitive and straightforward way to assess the accuracy of classification models for forestry pest detection. **Figure 13** shows the confusion matrix of the model before and after the improvement, where the rows and columns of the confusion matrix correspond to the proper and predicted categories, respectively. The values in the diagonal region indicate the proportion of correctly predicted categories, while the values in the other areas indicate the proportion of incorrectly predicted categories. The horizontal axis indicates the actual values, and the vertical axis indicates the expected values. The darker the color of the region, the more accurate the detection.

In comparison, it can be seen that there are five categories where the confidence level has improved significantly. Compared to YOLOv8, the improved confusion matrix has darker colors in the diagonal region, indicating a significant improvement in our model’s ability to predict object categories accurately. Our model achieved considerable performance improvements in most categories, but the confusion matrix shows that the recognition accuracy of some pest categories is still lower than expected. Possible reasons for these categories include the variability of lighting conditions in natural environments, such as strong sunlight, shadows, and reflections, which reduce image contrast, make pest features less prominent, lower recognition accuracy, and are related to decreased image quality under variable lighting conditions; The complex background of forestry environment, where elements such as leaves, branches, flowers, and fruits resemble pests in color and shape, makes it difficult for models to distinguish. Environmental elements may partially obscure pests, making it difficult for models to capture complete features, especially in images with occlusion.

### 4.4 Comparative experiments

In this paper, YOLOv3-tiny (due to the larger size of the original YOLOv3 model), YOLOv5, YOLOv7-tiny, YOLOv8, YOLOv9, and YOLOv10 of the YOLO family were compared. In comparing the YOLOv3-tiny model, Map@0.5:0.95(%) was reduced from 62.8 to 88.6 (25.8%), while the number of parameters was decreased from 8.73 to 1.91 (78%), computation was reduced from 13 to 5.2 (60%), and model size is compressed from 16.8 to 4.0 (76%). After the computation and pruning process, RSD-YOLOv8 was verified to be ahead of the other compared models in terms of detection accuracy, number of parameters, and model size. We also compared other target detection models. SSD and Faster R-CNN. Although the evaluation metrics of these models are comparable to RSD-YOLOv8. The significant computational complexity and the model size limit the feasibility of the above models. The weight size of RSD-YOLOv8 and the pruned model is only 0.7% of the Faster R-CNN model size. Inference time is also an important indicator for future deployment on mobile devices. RSD-YOLOv8, which has not undergone pruning, does not have a significant advantage in inference time compared to other models. RSD-YOLOv8 (1.5x), which has undergone pruning, simplifies the model structure by removing redundant or noncritical weights from the neural network, significantly decreasing inference time. However, YOLOv3 tiny and v5 have advantages in inference time, but they do not meet the practical needs of low parameters and high accuracy. The results of the comparison experiments are shown in **Table 7** below.

**Table 7:** Comparative data for different lightweight algorithms and v8 lightweight improvements.

References	Model	P (%)	R (%)	Map@0.5:0.95 (%)	Params (M)	FLOPs (G)	Weight Size (MB)	Inference time (ms)
Fuentes et al. (2017)	SSD	96.4	88.3	72.5	27.3	63.5	104	/
Shen et al. (2018)	Faster R-CNN	96.5	81.6	70.6	28.5	941.26	523	/
Wang et al. (2021)	YOLOv3-tiny	87.9	86.3	62.8	8.73	13.0	16.8	1.4
Li et al. (2022c)	YOLOv5	94.8	92.0	69.1	1.80	4.3	3.7	1.8
Jeong et al. (2022)	YOLOv7-tiny	93.9	94.1	73.0	6.08	13.3	11.9	2.4
Khalid et al. (2023)	YOLOv8	97.0	94.8	84.4	3.01	8.1	6.0	3.3
Wang et al. (2024)	YOLOv9	96.3	93.4	84.4	2.62	10.8	5.8	2.1
Liu et al. (2024)	YOLOv10	96.0	93.2	85.5	2.70	8.3	5.5	2.3
\	RSD-YOLOv8	97.6	94.6	86.0	2.61	7.6	5.4	3.1
\	RSD-YOLOv8 (1.5x)	98.0	95.6	88.6	1.91	5.2	4.0	1.9

**Figure 14:**
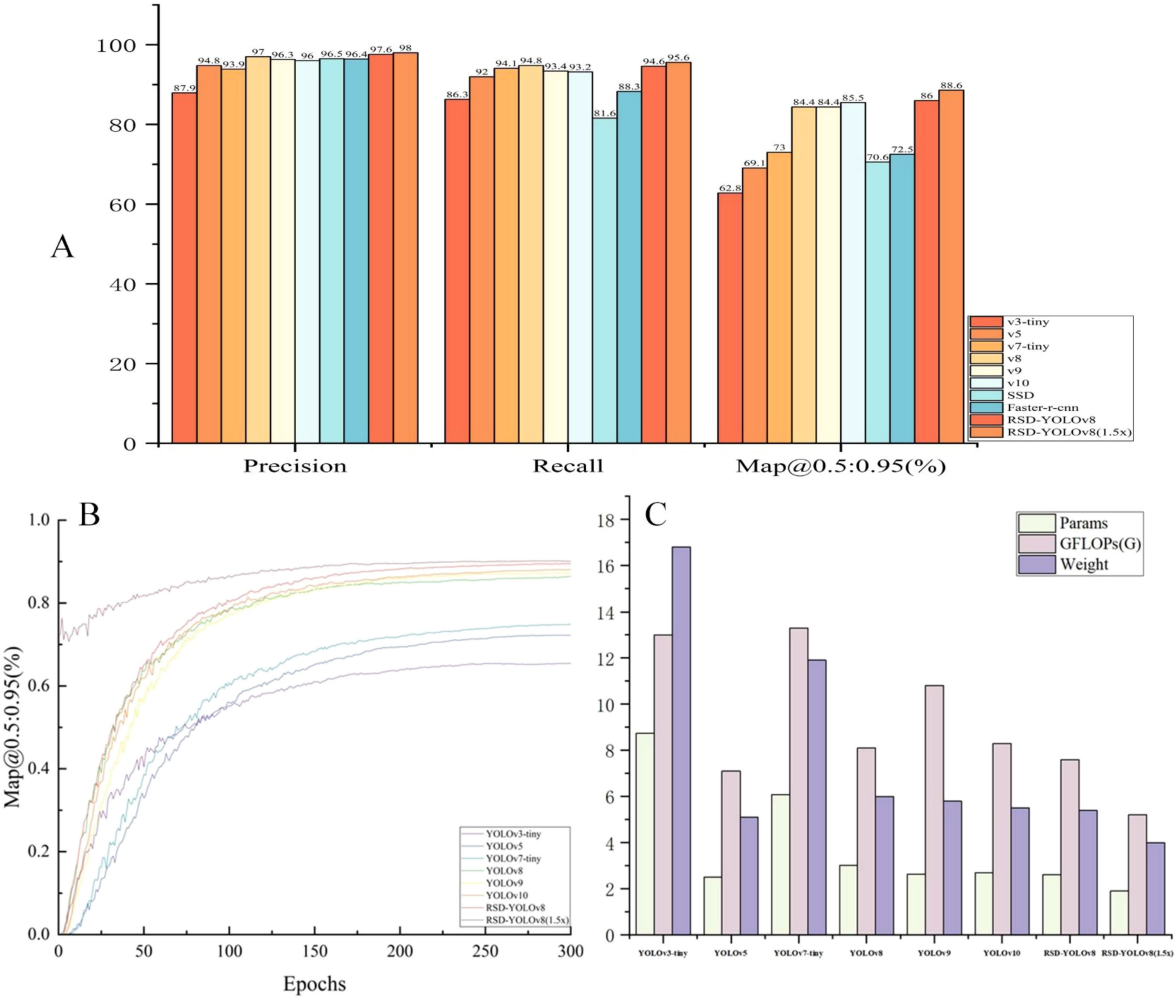
**(A)** Performance comparison of detection algorithms **(B)** Histogram of parameters versus operations and model size **(C)** shows a comparison of the Map@0.5:0.95(%) values of the training process of the YOLO series of models.

**Figure 14A** shows that RSD-YOLOv8, especially Map@0.5:0.95(%), an evaluation metric, has made significant progress. This result validates the proposed model’s effectiveness and highlights its potential advantages in practical applications. **Figure 14B** demonstrates that the proposed improved model is significantly smaller in scale than some of the comparative models, which gives our model a significant advantage in environments with limited storage and computational resources. In particular, when executing large-scale or real-time tasks, optimizing the model size can significantly reduce the hardware load and thus improve the execution efficiency, which opens up its possibilities in real-world application scenarios with resource constraints. The improved model significantly reduces model size through effective model design and pruning techniques while maintaining high performance. It gives the model a higher advantage in terms of computational and storage efficiency and makes it more convenient for handling large-scale data or real-time processing. **Figure 14C** clearly shows that the training curve is much higher than the training curves of the remaining models. It is also much lower than the size of the other compared models. The actual number and number of parameters are used to balance the accuracy, the amount of operations, and the number of parameters.

**Figure 15:**
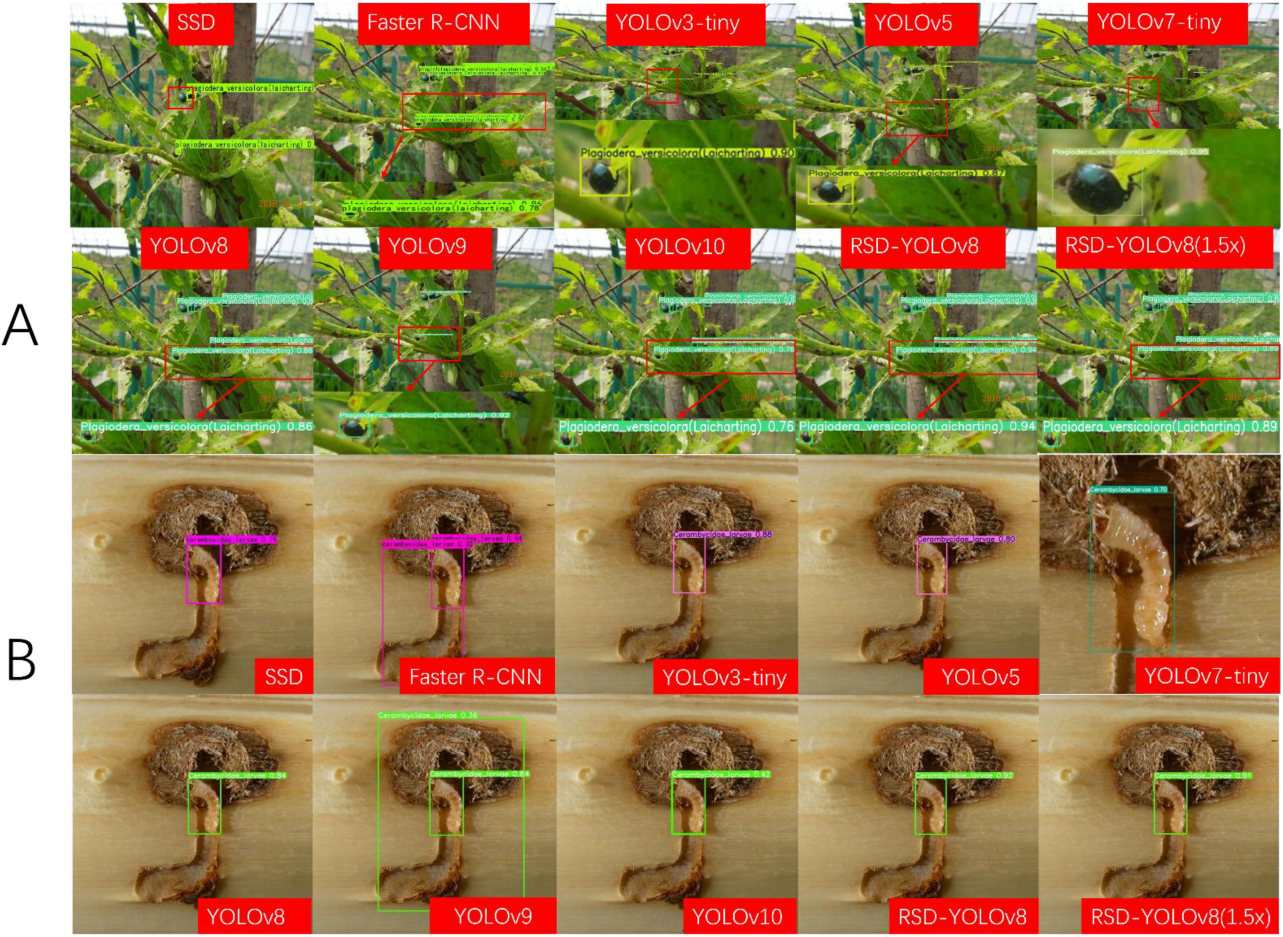
**(A)** Images of small target detection results for different models **(B)** Images of background and target similarity detection results for different models.

**Figure 15A** shows the detection results of the different models for the dense scene and occlusion tasks. Only YOLOv7-tiny has higher confidence than the improved model, but its computational and parametric complexity is three times higher than RSD-YOLOv8. **Figure 15B** shows the detection results of different models with similar detection targets and backgrounds. Among them, Faster R-CNN and YOLOv9 recognize the background error as a target. At the same time, RSD-YOLOv8 can solve problems such as false alarms and low accuracy to a certain extent and significantly improve prediction confidence. Considering the parameter and computational complexity of other models, RSD-YOLOv8 reduces the parameter and computational complexity and enhances the relative reliability to some extent. **Supplementary Table S1** shows the test data to compare the detection results of each model.

### 4.5 Model deployment and implementation

**Figure 16:**
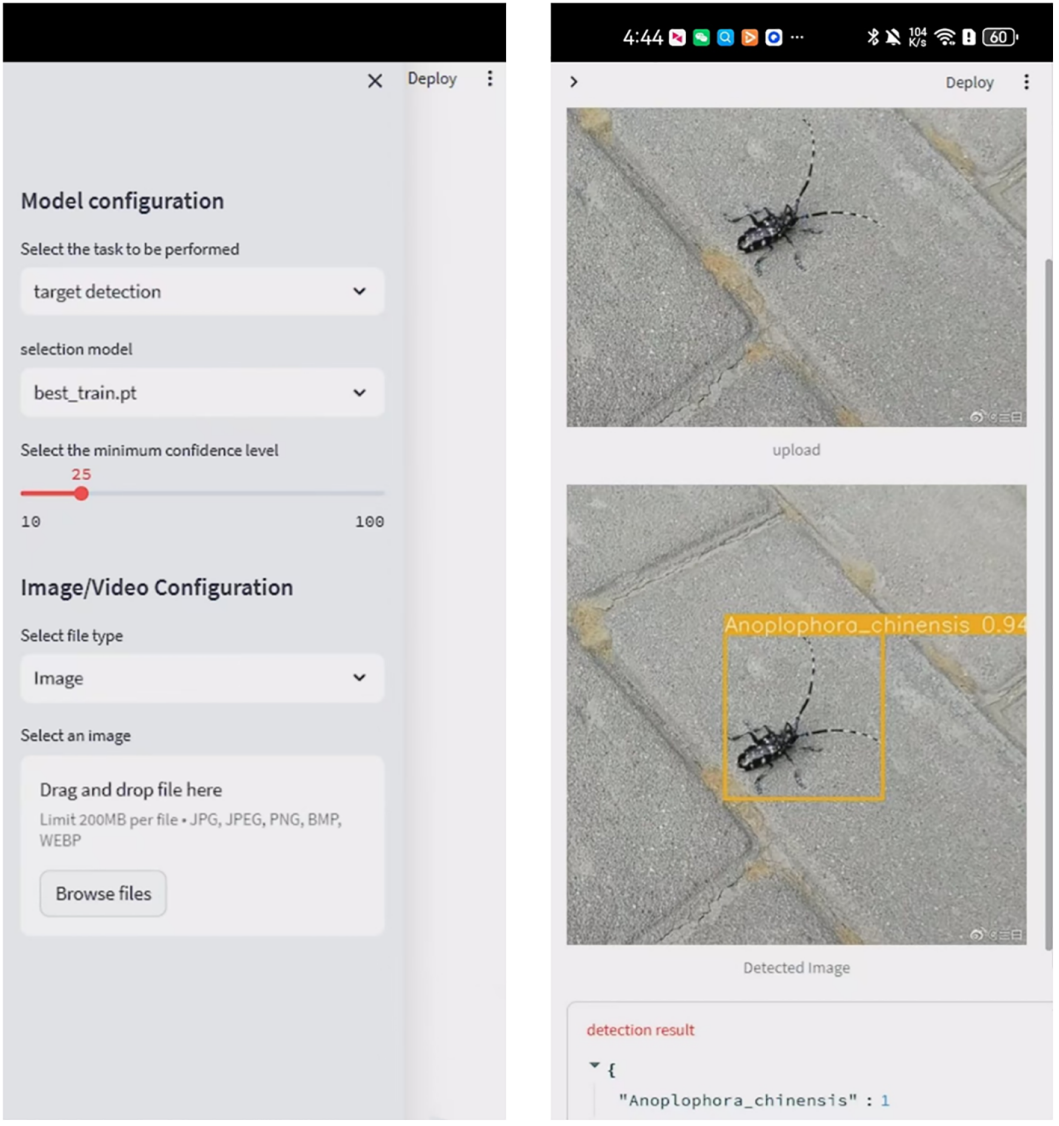
Mobile deployment detection results.

Developed a pest identification application based on the Android platform. Users can capture pest images through their mobile phone camera, and the application will call the local model in real-time for pest detection and display the recognition results. Multiple detection types (pictures, cameras, pictures) can be selected to verify that the model can realize real-time detection. The **Figure 16** shows the camera’s real-time detection results after deployment. The successful deployment on mobile devices provides important references for the application of the model on other embedded devices. Robots and still cameras are deployed in a similar way to mobile phones, relying on lightweight models and edge computing technology. The successful deployment on mobile devices validates the potential application of improved AI models on embedded devices. Based on the same technical framework, the model can quickly adapt to robots and static cameras, achieving automated monitoring and long-term data collection. In the future, we will continue to optimize model performance and explore deployment scenarios for more hardware devices to provide more efficient and accurate technical support for forestry pest detection.

## 5 Discussion

The proposed RSD-YOLOv8 model in this study addresses the shortcomings of deep learning models in dealing with large-scale multicategory forestry pests. By combining RepHGNetv2, Slim-neck, and Dyhead modules, in which the regular convolution of HGBlock in HGNetv2 in RepHGNetv2 is replaced by the proposed RepLightConv, which maintains a high model expressiveness and simultaneously reduces the number of parameters and computation. The Neck part uses the Slim-Neck module. GSConv and VoVGSCSP preserve as many hidden connections of these channels as possible. The purpose of the appealing improvements is to address resource constraints and computational limitations for deployment in forestry pest detection tasks. The detection head DyHead, which includes an attention mechanism, is introduced to gradually extract information from the feature map through scale, spatial, and task awareness. Adaptively adjust the size of perception to adapt to the scale change of different targets and improve the detection ability of targets at various scales. Better realization of small target pest feature information extraction.

**Figure 17:**
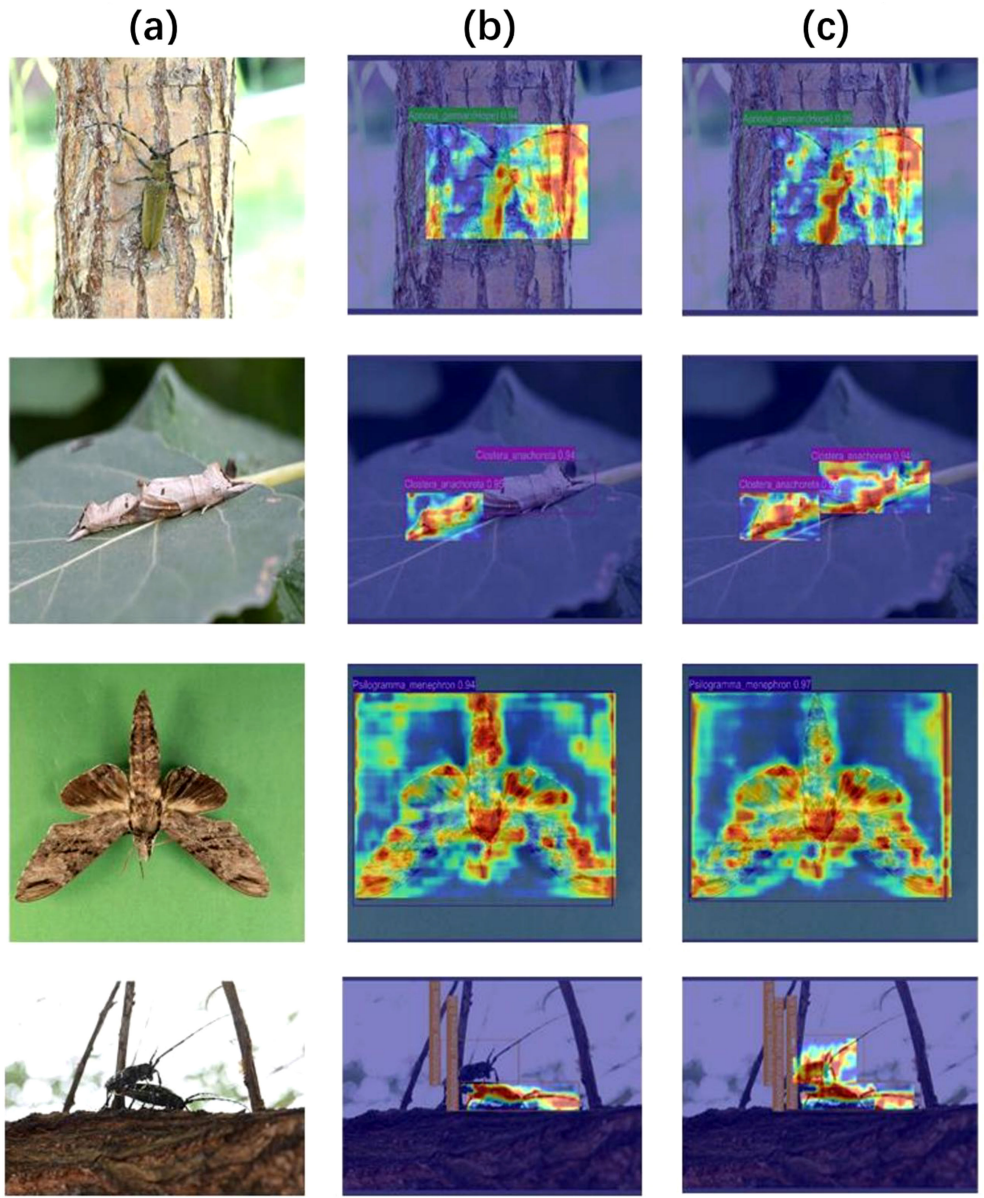
**(a)** Original image, **(b)** YOLOv8 **(c)** RSD-YOLOv8.

The superior performance of RSD-YOLOv8 is verified through ablation and comparison experiments, and the results are significantly better than YOLOv8 and its mainstream models. The advantages of RSD-YOLOv8 lie in the replacement of the backbone network by YOLOv8, the lightweight of the neck, and the improvement of the detection head. The model’s effectiveness was visually evaluated using the Grad CAM (Selvaraju et al., 2017) technique, and **Figure 17** compares the thermogram results before and after the improvements. It is demonstrated that RSD-YOLOv8 focuses more on localized areas of the input feature map, enhancing the model’s ability to understand and capture spatial details.

Future research will combine optimization strategies of knowledge distillation (Zhang et al., 2024) and model pruning techniques, aiming to enhance further the performance of the RSD-YOLOv8 model for small target pest detection. We hope to achieve lightweight models by combining these technologies while maintaining or improving the model’s generalization ability and detection accuracy. The core of this optimization strategy lies in transferring knowledge from larger and more complex models to smaller and more efficient models through knowledge distillation, in order to optimize detection performance. The forestry pest detection system has made certain progress. Therefore, our research objective will be to develop mobile device applications based on actual application needs. This method provides valuable guidance for the development of portable mobile device terminals and offers powerful and practical solutions for the fields of forestry and plant protection. By deploying this technology in embedded devices, our goal is to promote the development of forest pest detection technology and provide more efficient and accurate technical support for forest management. In order to cope with the popularity of unmanned aerial vehicles in intelligent forestry, attempts have been made to deploy models on unmanned aerial vehicles. However, for forestry pest detection tasks, especially for small target pests, traditional visible light cameras may not provide sufficient resolution to capture the details of small targets. Hyperspectral images have narrower and more spectral bands and have been applied to monitor forest pests. The resolution of visible light cameras is relatively low, which may mask subtle changes caused by pests. Therefore, we plan to explore the combination of models with hyperspectral cameras or multispectral imaging techniques in future research to improve the detection capability of small target pests. We plan to combine target detection technology with multispectral and hyperspectral imaging technology in future research and deploy it on unmanned aerial vehicle platforms to achieve more flexible large-scale monitoring and richer spectral information acquisition, thereby improving the accuracy and robustness of pest detection. In future experiments, we will focus on the impact of the distance between the camera and the target (such as 2 to 20 meters) on image quality and detection accuracy, and optimize camera parameters to meet different scene requirements; Simultaneously testing the image acquisition performance of drones at different flight speeds (such as 1 meter/s to 5 meters/s), analyzing the impact of speed on target detection accuracy, and quantifying the relationship between speed and accuracy through experiments, exploring technical solutions to maintain high accuracy under high-speed movement conditions (such as motion blur compensation algorithms). This research direction will significantly enhance the practicality and scalability of pest monitoring technology, laying a solid foundation for future practical applications. Hyperspectral cameras can capture richer spectral information, which helps to distinguish pests from the background environment and improve detection accuracy.

## 6 Conclusions

In this study, we made innovative modifications based on the YOLOv8 architecture, including introducing RepLightConv convolution and Slim neck module to simplify the network structure and integrating the Dyhead detection head to improve RSD-YOLOv8. The comparative analysis with existing lightweight networks and various object detection algorithms further confirms the superior performance of RSD-YOLOv8, among which RSD-YOLOv8 outperforms YOLOv8n in terms of performance Map@0.50.95 The accuracy has reached 88.6%, the number of parameters has been reduced to 1.9M, which is 36% lower than the original model. The computational complexity has been reduced by 36%, and the model size has been reduced by 33%. These improvements make our model more suitable for forestry pest detection, especially when dealing with large-scale and multi-species detection tasks, exhibiting high accuracy and low parameter characteristics.

More importantly, this work improves the algorithm’s performance and has profound significance in practical applications. In resource-constrained environments such as remote forest areas, the low-parameter and high-precision characteristics of RSD-YOLOv8 are particularly critical. It can significantly reduce the demand for computing resources without sacrificing detection performance, a revolutionary progress for forest conservation management. Our model can be more easily deployed on mobile devices or drones, enabling real-time and efficient pest monitoring, which is crucial for early detection and prevention of pest infestations. In addition, by reducing reliance on expensive hardware and mighty computing power, RSD-YOLOv8 greatly reduces the cost of forest conservation management, making advanced pest detection technologies more widespread and feasible.

Therefore, RSD-YOLOv8 shows its progressiveness in academia and has essential application potential and value in actual forest pest detection. It is expected to completely change the status of forest protection and management and provide strong technical support for sustainable forest health management.

We believe that the framework and optimization strategies adopted by RSD-YOLOv8 are equally applicable to object detection tasks in other fields. For example, the model can be extended to multiple fields, such as agricultural pest detection, wildlife monitoring, and object recognition in urban environments. Through further research and adaptation, RSD-YOLOv8 has the potential to become a multifunctional detection tool, providing efficient and real-time monitoring solutions for different industries, thereby promoting the application of intelligent detection technology in a broader range of environmental protection and resource management.

## Data Availability

The original contributions presented in the study are included in the article/**Supplementary Material**. Further inquiries can be directed to the corresponding authors.

## References

[B1] Al-Hiary H. Bani-Ahmad S. Reyalat M. Braik M. Alrahamneh Z. (2011). Fast and accurate detection and classification of plant diseases. Int. J. Comput. Appl. 17, 31–38. doi: 10.5120/2183-2754

[B2] Bongaarts J. (2019). IPBES 2019. Summary for policymakers of the global assessment report on biodiversity and ecosystem services of the Intergovernmental Science-Policy Platform on Biodiversity and Ecosystem Services. Popul. Dev. Rev. 45, 680–681. doi: 10.1111/padr.12283

[B3] Checola G. Sonego P. Zorer R. Mazzoni V. Ghidoni F. Gelmetti A.. (2024). A novel dataset and deep learning object detection benchmark for grapevine pest surveillance. Front. Plant Sci. 15. doi: 10.3389/fpls.2024.1485216 PMC11669504 39726421

[B4] Chen H. Wang Y. Guo J. Tao D. (2023a). VanillaNet: the power of minimalism in deep learning. arXiv preprint arXiv:2305.12972. doi: 10.48550/arXiv.2305.12972

[B5] Chen H. Zhou G. Jiang H. (2023b). Student behavior detection in the classroom based on improved YOLOv8. Sensors 23, 8385. doi: 10.3390/s23208385 37896479 PMC10611206

[B6] Cheng Z. K. Huang R. Q. Qian R. Dong W. Zhu J. B. Liu M. F. (2022). A lightweight crop pest detection method based on convolutional neural networks. Appl. Sci.-Basel 12, 21. doi: 10.3390/app12157378

[B7] Choi W. I. Park Y.-S. (2019). Monitoring, assessment and management of forest insect pests and diseases. Forests 10, 865. doi: 10.3390/f10100865

[B8] Dai X. Chen Y. Xiao B. Chen D. Liu M. Yuan L.. (2021). “Dynamic head: unifying object detection heads with attentions,” in 2021 IEEE/CVF Conference on Computer Vision and Pattern Recognition (CVPR), Nashville, TN, USA: IEEE. 7369–7378. doi: 10.1109/CVPR46437.2021.00729

[B9] Ding X. Zhang X. Ma N. Han J. Ding G. Sun J. (2021). “Repvgg: Making vgg-style convnets great again,” in 2021 IEEE/CVF Conference on Computer Vision and Pattern Recognition (CVPR), Nashville, TN, USA: IEEE. 13733–13742. doi: 10.1109/CVPR46437.2021.01352

[B10] Dong Y. Y. Huang W. J. Lin K. J. Han L. X. Laneve G. Zhang J. C. (2024). Editorial: Pests and diseases monitoring and forecasting algorithms, technologies, and applications. Front. Plant Sci. 15. doi: 10.3389/fpls.2024.1518814 PMC11654248 39698451

[B11] Duan E. Han G. Zhao S. Ma Y. Lv Y. Bai Z. (2023). Regulation of meat duck activeness through photoperiod based on deep learning. Animals 13, 3520. doi: 10.3390/ani13223520 38003138 PMC10668642

[B12] Fuentes A. Yoon S. Kim S. C. Park D. S. (2017). A robust deep-learning-based detector for real-time tomato plant diseases and pests recognition. Sensors 17, 21. doi: 10.3390/s17092022 PMC5620500 28869539

[B13] Howard A. Sandler M. Chu G. Chen L.-C. Chen B. Tan M.. (2019). “Searching for mobilenetv3,” in 2019 IEEE/CVF International Conference on Computer Vision (ICCV), Seoul, Korea South: IEEE. 1314–1324. doi: 10.1109/ICCV.2019.00140

[B14] Huang L. Chen J. Li H. Huang Y. She K. Hao K. (2024). Excellent tomato detector based on pruning and distillation to balance accuracy and lightweight. Comput. Electron. Agric. 227, 109520. doi: 10.1016/j.compag.2024.109520

[B15] Jeong Y. Hwang H.-S. Kwon Y. J. Lee C.-H. (2022). Design and implementation of real-time wasp monitoring system using edge device. J. Korea Multimed. Soc. 25, 1826–1839. doi: 10.9717/kmms.2022.25.12.1826

[B16] Jiang T. Chen S. (2024). A lightweight forest pest image recognition model based on improved YOLOv8. Appl. Sci. 14, 1941. doi: 10.3390/app14051941

[B17] Khalid S. Oqaibi H. M. Aqib M. Hafeez Y. (2023). Small pests detection in field crops using deep learning object detection. Sustainability 15, 19. doi: 10.3390/su15086815

[B18] Lee J. Park S. Mo S. Ahn S. Shin J. (2020). Layer-adaptive sparsity for the magnitude-based pruning. arXiv preprint arXiv:2010.07611. doi: 10.48550/arXiv.2010.07611

[B19] Li D. Ahmed F. Wu N. Sethi A. I. (2022a). Yolo-JD: A Deep Learning Network for jute diseases and pests detection from images. Plants 11, 937. doi: 10.3390/plants11070937 35406915 PMC9003326

[B20] Li H. Li J. Wei H. Liu Z. Zhan Z. Ren Q. (2022b). Slim-neck by GSConv: A better design paradigm of detector architectures for autonomous vehicles. arXiv preprint arXiv:2206.02424. doi: 10.1007/s11554-024-01436-6

[B21] Li W. Zhu T. F. Li X. Y. Dong J. Z. Liu J. (2022c). Recommending advanced deep learning models for efficient insect pest detection. Agriculture-Basel 12, 17. doi: 10.3390/agriculture12071065

[B22] Lin T.-Y. Dollár P. Girshick R. He K. Hariharan B. Belongie S. (2017). “Feature pyramid networks for object detection,” in 2017 IEEE Conference on Computer Vision and Pattern Recognition (CVPR), Honolulu, HI, USA: IEEE. 2117–2125. doi: 10.1109/CVPR.2017.106

[B23] Liu W. Anguelov D. Erhan D. Szegedy C. Reed S. Fu C.-Y.. (2016). “SSD: single shot multiBox detector,” in Computer Vision – ECCV 2016, Amsterdam, Netherlands: Springer. 21–37. doi: 10.1007/978-3-319-46448-0_2

[B24] Liu B. Jia Y. Liu L. Dang Y. Song S. (2023). Skip DETR: end-to-end Skip connection model for small object detection in forestry pest dataset. Front. Plant Sci. 14. doi: 10.3389/fpls.2023.1219474 PMC10464905 37649993

[B25] Liu B. Liu L. Zhuo R. Chen W. Duan R. Wang G. (2022). A dataset for forestry pest identification. Front. Plant Sci. 13. doi: 10.3389/fpls.2022.857104 PMC9331284 35909784

[B26] Liu S. Qi L. Qin H. Shi J. Jia J. (2018). “Path aggregation network for instance segmentation,” in 2018 IEEE/CVF Conference on Computer Vision and Pattern Recognition, Salt Lake City, UT, USA: IEEE. 8759–8768. doi: 10.1109/CVPR.2018.00913

[B27] Liu J. Wang X. (2021). Plant diseases and pests detection based on deep learning: a review. Plant Methods 17, 1–18. doi: 10.1186/s13007-021-00722-9 33627131 PMC7903739

[B28] Liu Y. K. Wang Q. H. Zheng Q. Liu Y. (2024). YOLO-wheat: A more accurate real-time detection algorithm for wheat pests. Agriculture-Basel 14, 17. doi: 10.3390/agriculture14122244

[B29] Ma X. Dai X. Bai Y. Wang Y. Fu Y. (2024). Rewrite the stars. arXiv preprint arXiv:2403.19967 5694-5703. doi: 10.48550/arXiv.2403.19967

[B30] Qin D. Leichner C. Delakis M. Fornoni M. Luo S. Yang F.. (2025). “MobileNetV4: universal models for the mobile ecosystem,” in Computer Vision – ECCV 2024, Milan, Italy: Springer. 78–96. doi: 10.1007/978-3-031-73661-2_5

[B31] Redmon J. Divvala S. Girshick R. Farhadi A. (2016). “You only look once: Unified, real-time object detection,” in 2016 IEEE Conference on computer vision and Pattern recognition, Las Vegas, NV, USA: IEEE. 779–788. doi: 10.1109/CVPR.2016.91

[B32] Ren S. He K. Girshick R. Sun J. (2016). Faster R-CNN: Towards real-time object detection with region proposal networks. IEEE Trans. Pattern Anal. Mach. Intell. 39, 1137–1149. doi: 10.1109/TPAMI.2016.2577031 27295650

[B33] Selvaraju R. R. Cogswell M. Das A. Vedantam R. Parikh D. Batra D. (2017). “Grad-cam: Visual explanations from deep networks via gradient-based localization”, in 2017 IEEE International Conference on Computer Vision (ICCV), Venice, Italy: IEEE, 618–626. doi: 10.1109/ICCV.2017.74

[B34] Shen L. Y. Lang B. H. Song Z. X. (2023). Infrared object detection method based on DBD-YOLOv8. IEEE Access 11, 145853–145868. doi: 10.1109/access.2023.3345889

[B35] Shen Y. F. Zhou H. L. Li J. T. Jian F. J. Jayas D. S. (2018). Detection of stored-grain insects using deep learning. Comput. Electron. Agric. 145, 319–325. doi: 10.1016/j.compag.2017.11.039

[B36] Wang X. W. Liu J. Liu G. X. (2021). Diseases detection of occlusion and overlapping tomato leaves based on deep learning. Front. Plant Sci. 12. doi: 10.3389/fpls.2021.792244 PMC8702556 34956290

[B37] Wang Z. J. Zhang S. H. Chen L. J. Wu W. D. Wang H. Q. Liu X. H.. (2024). Microscopic insect pest detection in tea plantations: improved YOLOv8 model based on deep learning. Agriculture-Basel 14, 21. doi: 10.3390/agriculture14101739

[B38] Wen C. Chen H. Ma Z. Zhang T. Yang C. Su H.. (2022). Pest-YOLO: A model for large-scale multi-class dense and tiny pest detection and counting. Front. Plant Sci. 13. doi: 10.3389/fpls.2022.973985 PMC9783619 36570910

[B39] Wu X. Zhan C. Lai Y.-K. Cheng M.-M. Yang J. (2019). “Ip102: A large-scale benchmark dataset for insect pest recognition,” in 2019 IEEE/CVF Conference on Computer Vision and Pattern Recognition (CVPR), Long Beach, CA, USA: IEEE. 8787–8796. doi: 10.1109/CVPR.2019.00899

[B40] Xiang Q. Huang X. Huang Z. Chen X. Cheng J. Tang X. (2023). YOLO-pest: an insect pest object detection algorithm via CAC3 module. Sensors 23, 3221. doi: 10.3390/s23063221 36991930 PMC10059078

[B41] Yang Z.-Q. Wang X.-Y. Zhang Y.-N. (2014). Recent advances in biological control of important native and invasive forest pests in China. Biol. Control 68, 117–128. doi: 10.1016/j.biocontrol.2013.06.010

[B42] Yang S. Xing Z. Wang H. Dong X. Gao X. Liu Z.. (2023). Maize-YOLO: a new high-precision and real-time method for maize pest detection. Insects 14, 278. doi: 10.3390/insects14030278 36975962 PMC10051432

[B43] Yu J. Zhang B. (2023). Mdp-yolo: A lightweight yolov5s algorithm for multi-scale pest detection. Engenharia Agrícola 43, 16. doi: 10.1590/1809-4430-Eng.Agric.v43n4e20230065/2023

[B44] Zeng T. H. Li S. Y. Song Q. M. Zhong F. L. Wei X. (2023). Lightweight tomato real-time detection method based on improved YOLO and mobile deployment. Comput. Electron. Agric. 205, 14. doi: 10.1016/j.compag.2023.107625

[B45] Zhang L. Ding G. Li C. Li D. (2023a). DCF-Yolov8: an improved algorithm for aggregating low-level features to detect agricultural pests and diseases. Agronomy 13, 2012. doi: 10.3390/agronomy13082012

[B46] Zhang M. Y. Gao F. Yang W. P. Zhang H. R. (2023b). Wildlife object detection method applying segmentation gradient flow and feature dimensionality reduction (vol 12, 377, 2023). Electronics 12, 1. doi: 10.3390/electronics12081923

[B47] Zhang X. L. Liang K. Zhang Y. Y. (2024). Plant pest and disease lightweight identification model by fusing tensor features and knowledge distillation. Front. Plant Sci. 15. doi: 10.3389/fpls.2024.1443815 PMC11617168 39640993

[B48] Zhao J. Wang J. Huang J. Zhang L. Tang J. (2023). Spring temperature accumulation is a primary driver of forest disease and pest occurrence in China in the context of climate change. Forests 14, 1730. doi: 10.3390/f14091730

[B49] Zhao Y. Lv W. Xu S. Wei J. Wang G. Dang Q.. (2024). “Detrs beat yolos on real-time object detection,” in 2024 IEEE/CVF Conference on Computer Vision and Pattern Recognition (CVPR), Seattle, WA, USA: IEEE. 16965–16974. doi: 10.1109/CVPR52733.2024.01605

