# Supplementary material for: YOLOv8 forestry pest recognition based on improved re-parametric convolution
Source: Front Plant Sci. 2025 Mar 11;16:1552853. doi: 10.3389/fpls.2025.1552853 (PMC11933051; doi:10.3389/fpls.2025.1552853)
Supplement: Supplementary file 1 [file DataSheet1.docx]

Supplementary Material

| RSD-YOLOv8 | 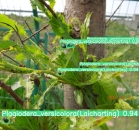 | 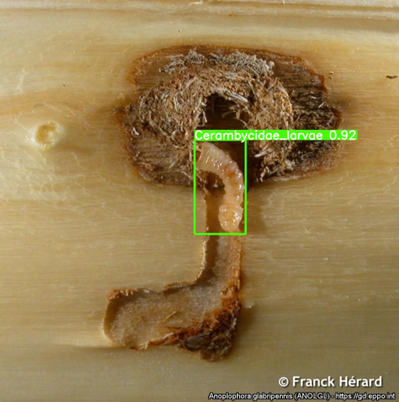 | 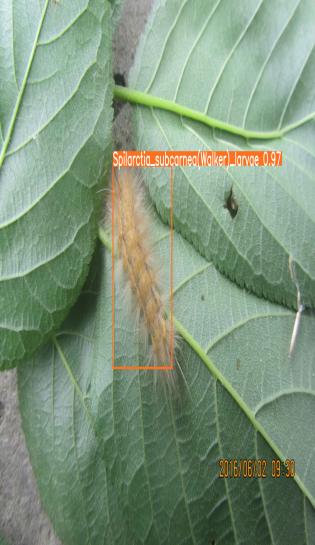 | 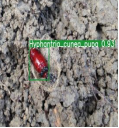 | 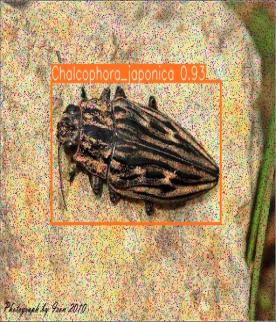 | 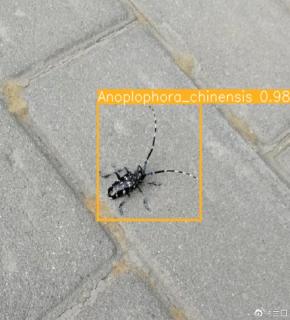 |
| --- | --- | --- | --- | --- | --- | --- |
| RSD-YOLOv8(1.5x) | 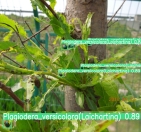 | 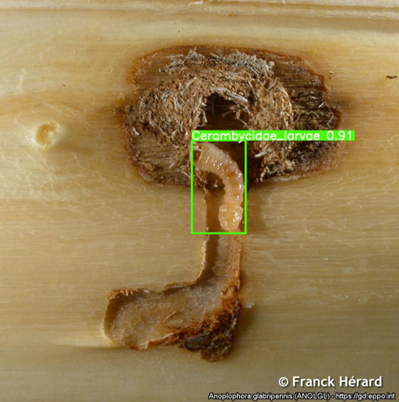 | 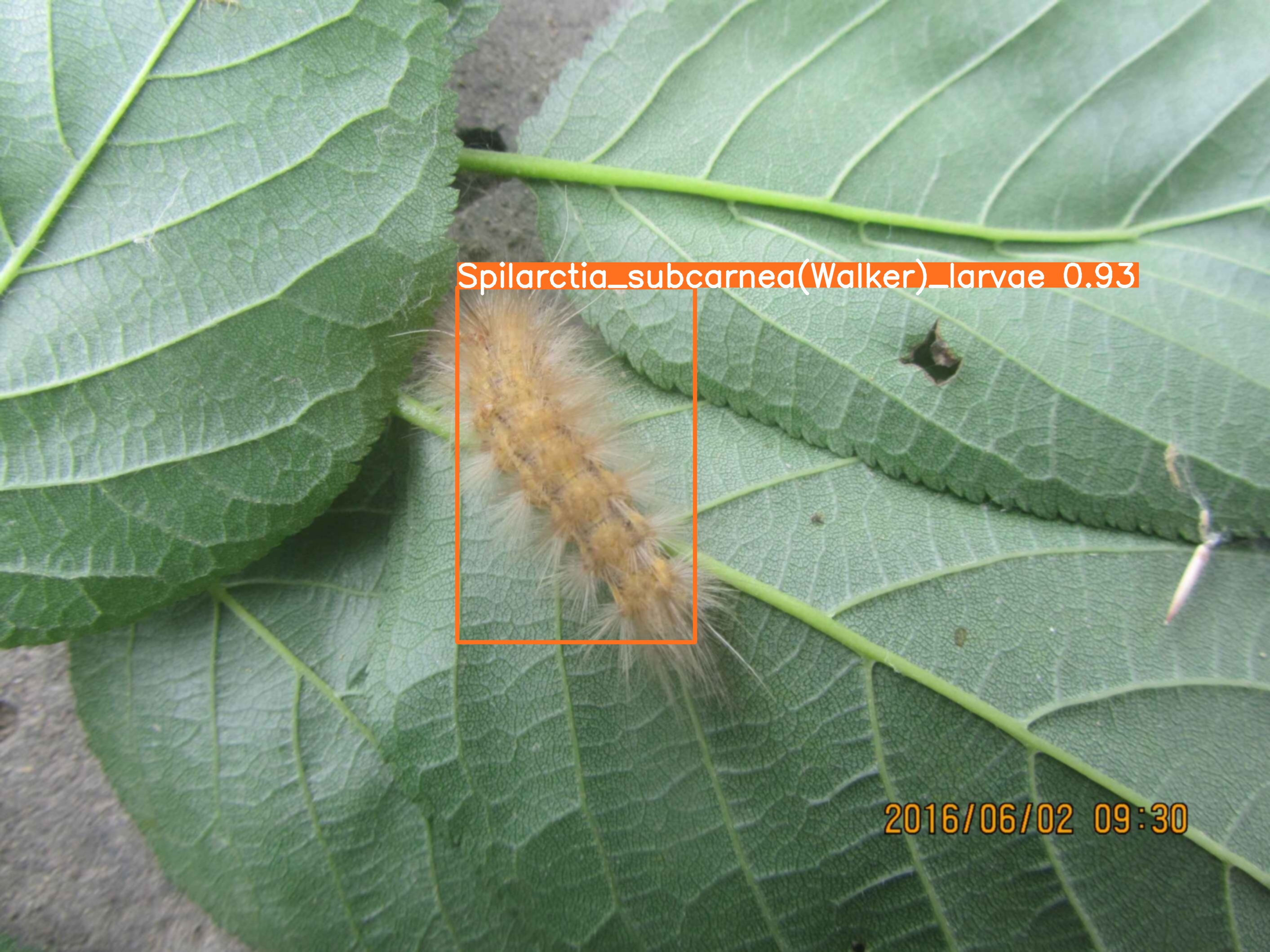 | 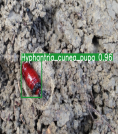 | 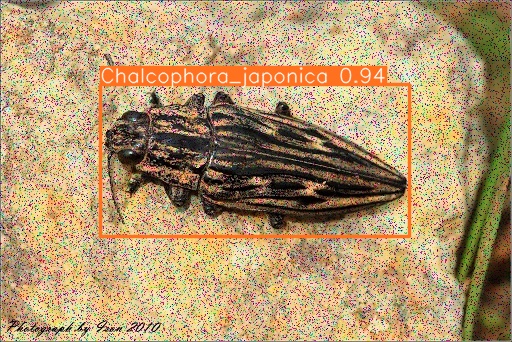 | 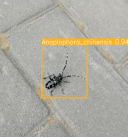 |
| YOLOv8 | 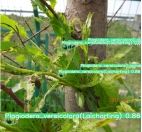 | 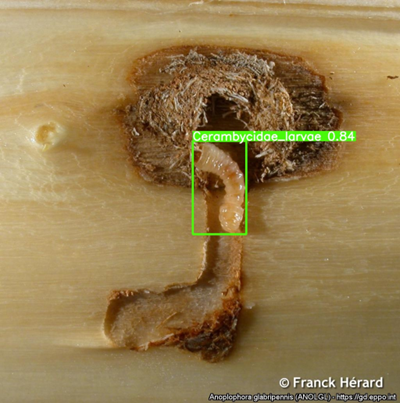 | 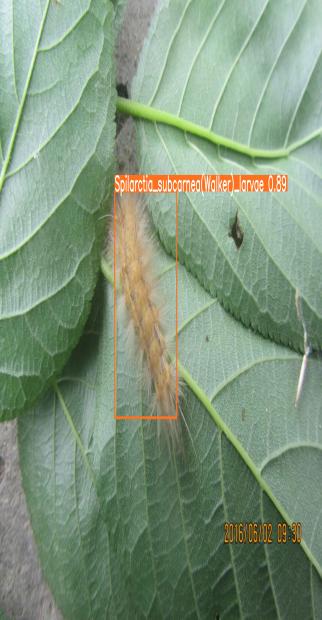 | 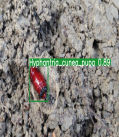 | 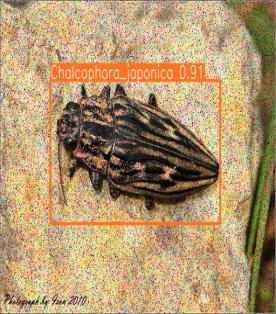 | 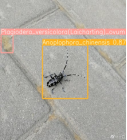 |
| YOLOv10 | 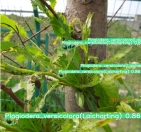 | 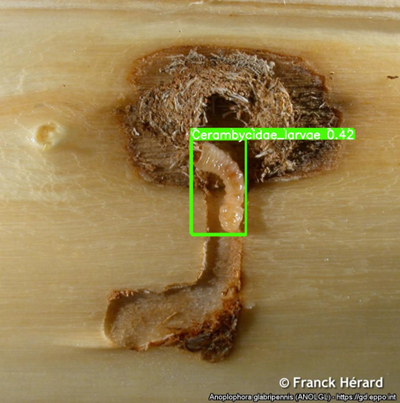 | 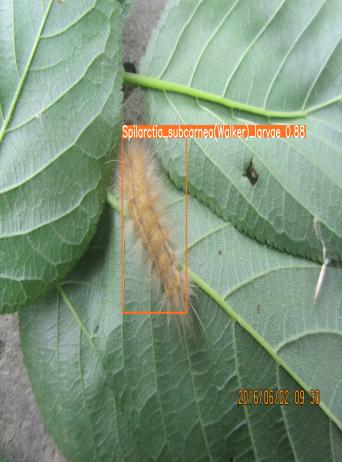 | 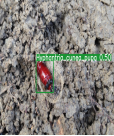 | 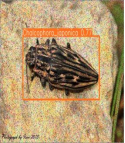 | 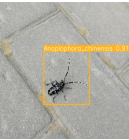 |
| YOLOv9 | 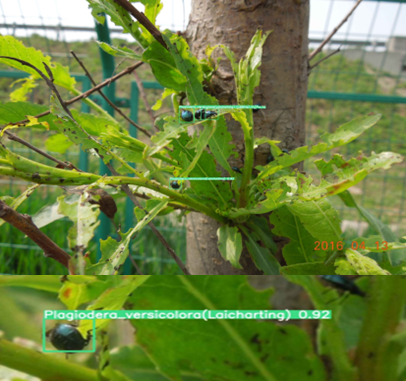 | 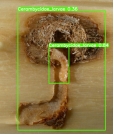 | 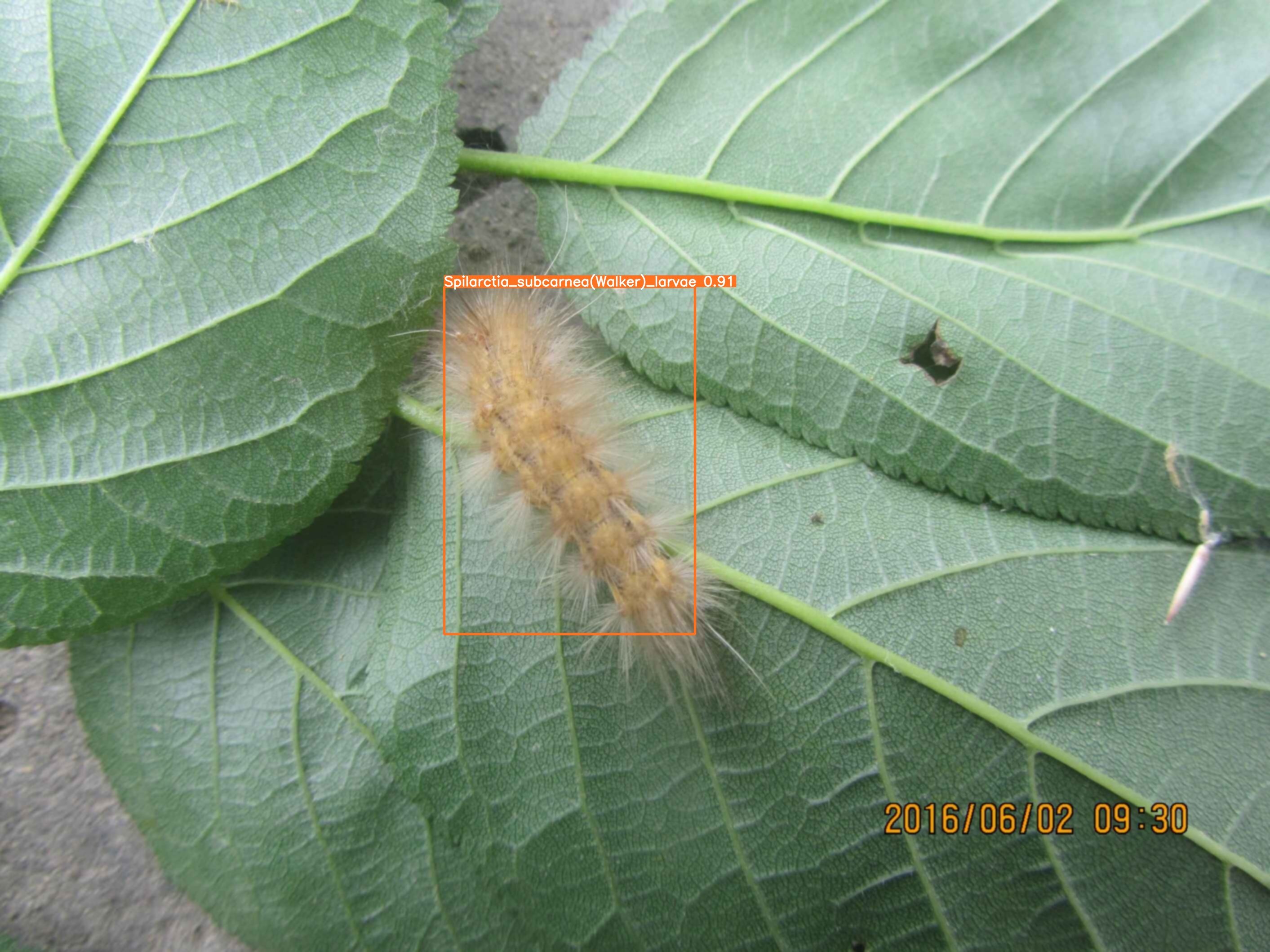 | 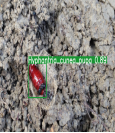 | 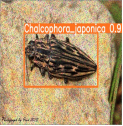 | 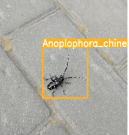 |
| YOLOv5 | 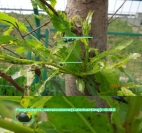 | 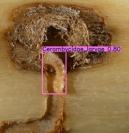 | 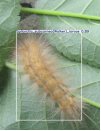 | 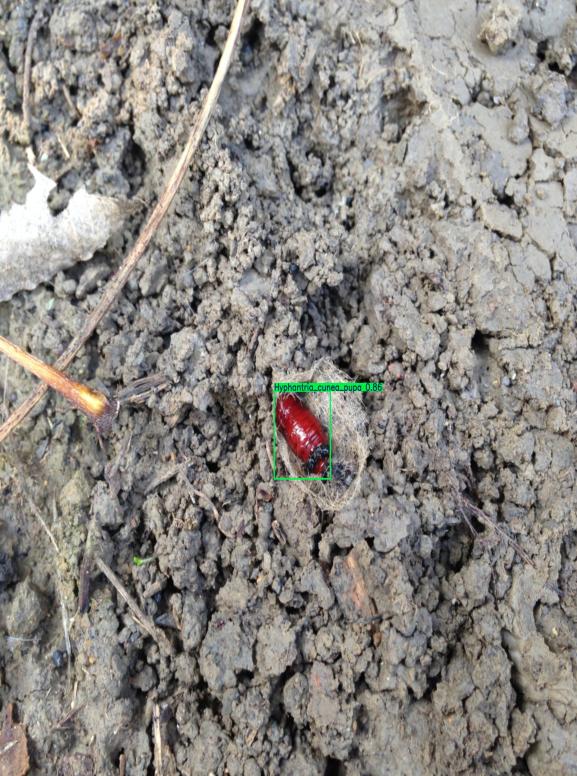 | 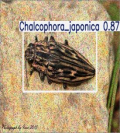 | 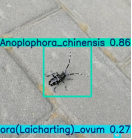 |
| YOLOv7-tiny | 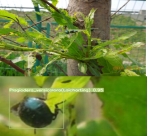 | 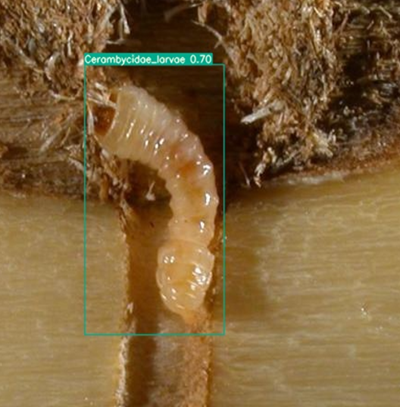 | 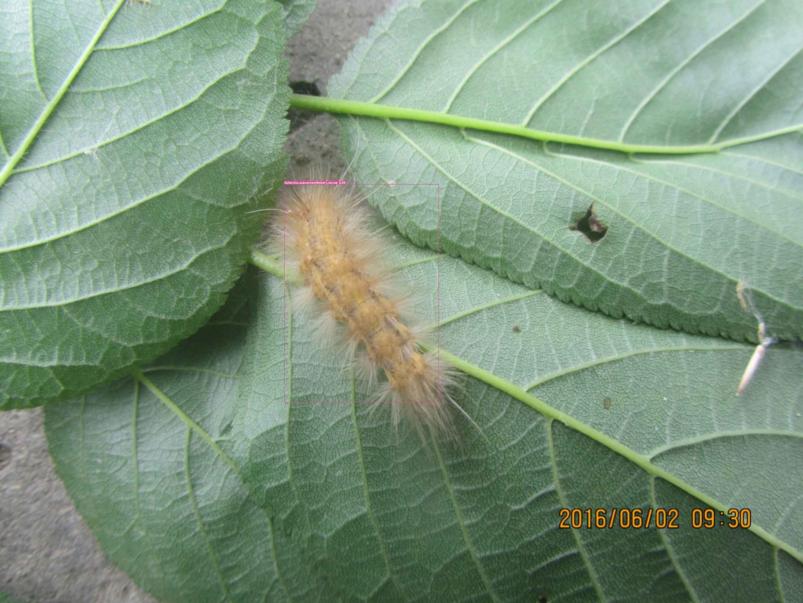 | 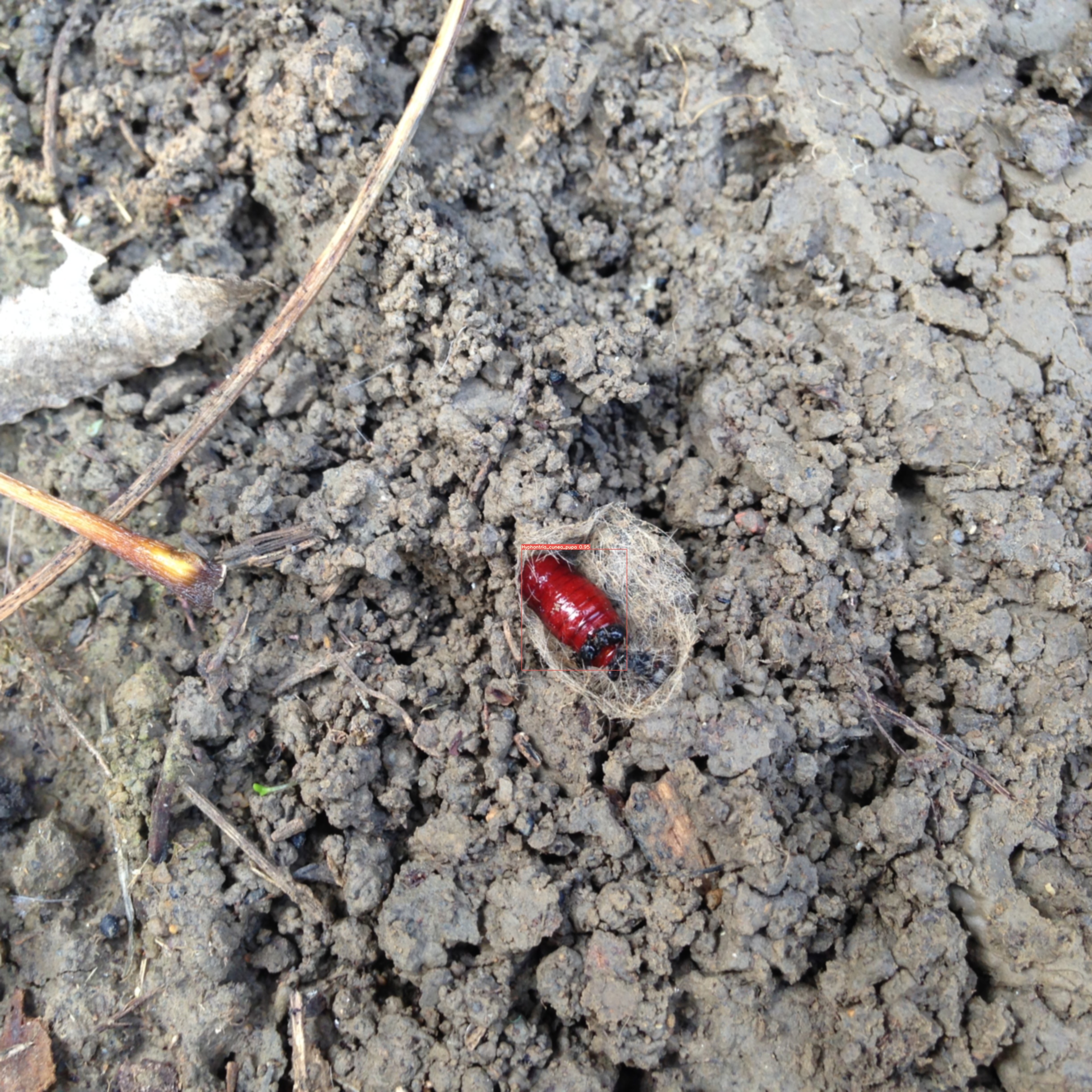 | 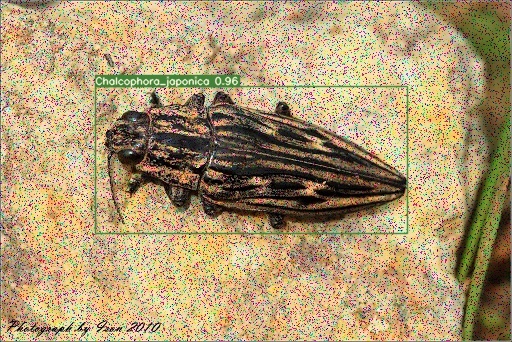 | 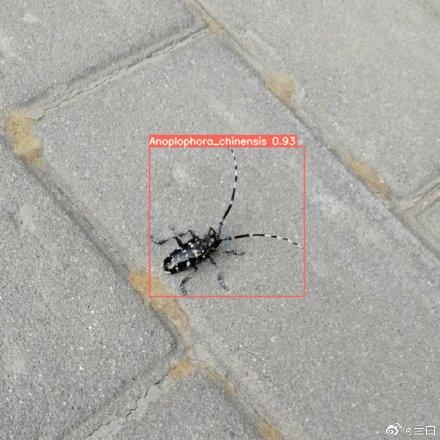 |
| YOLOv3-tiny | 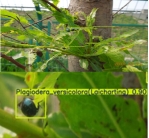 | 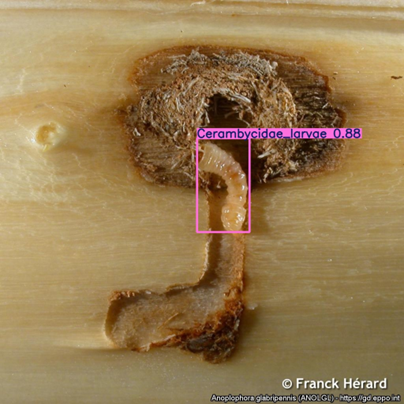 | 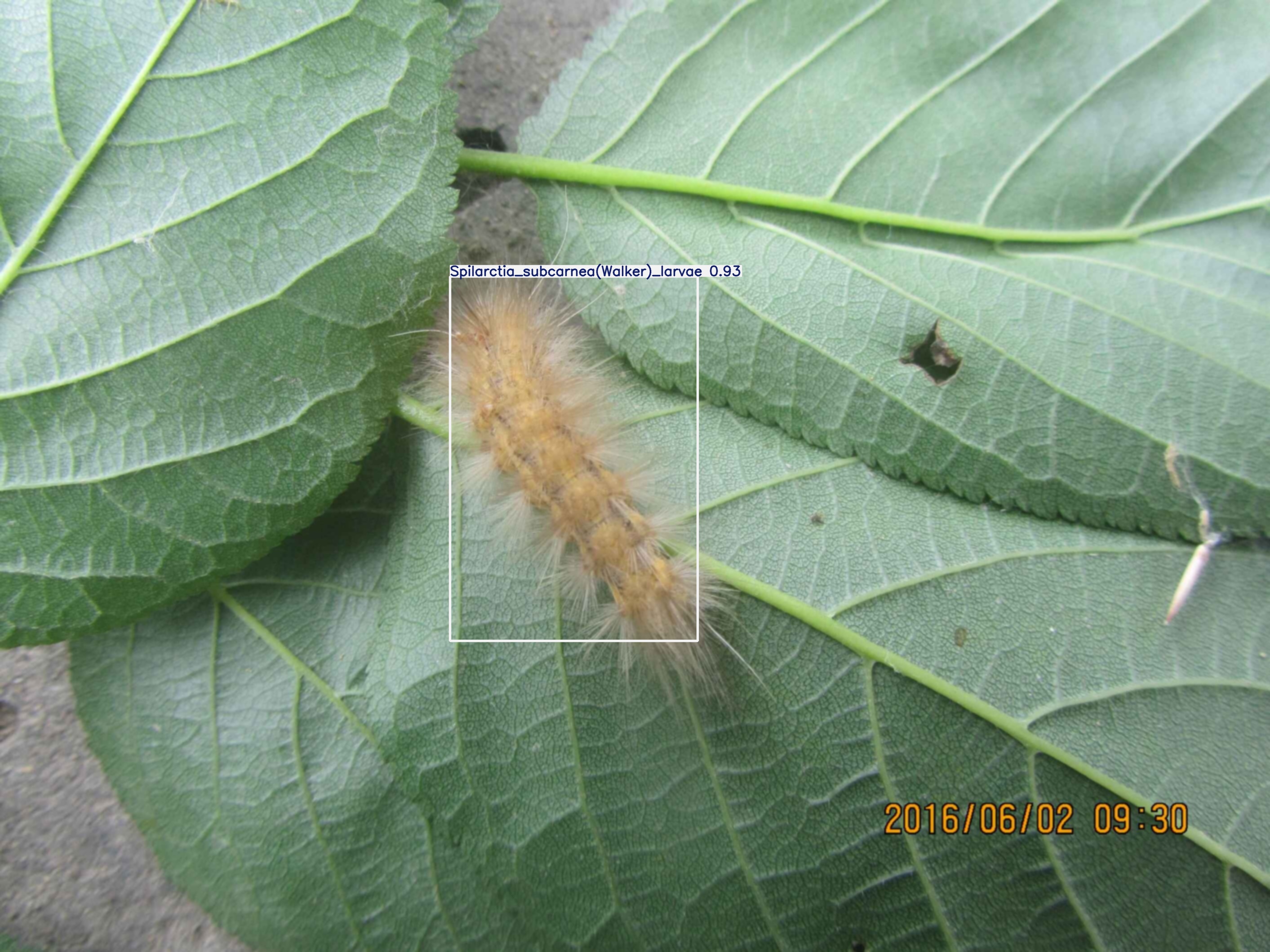 | 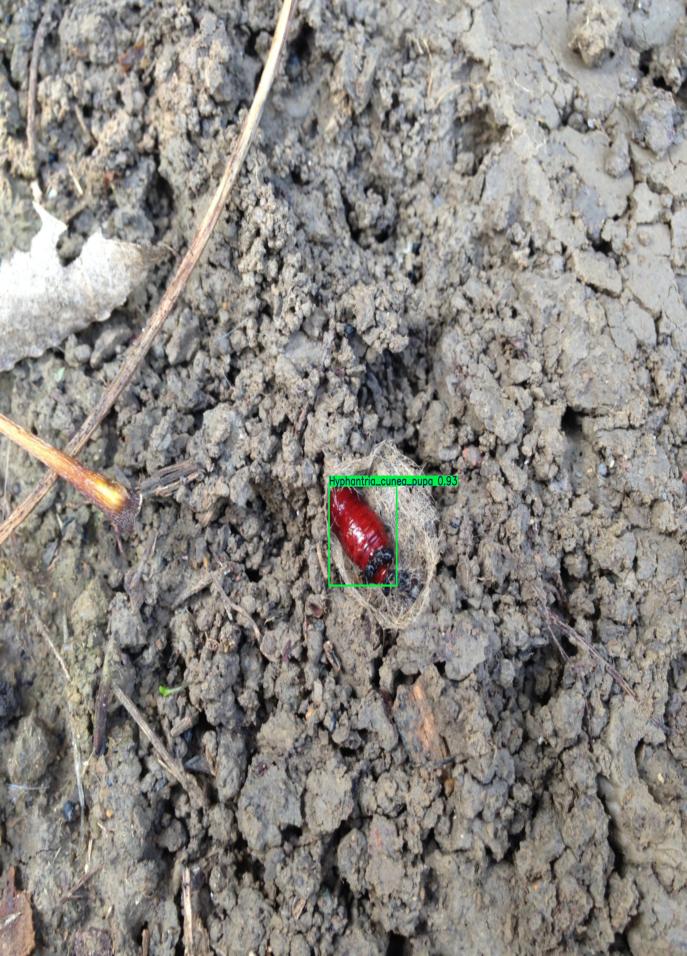 | 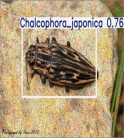 | 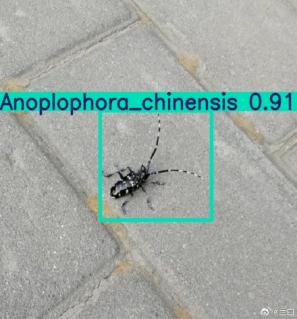 |
| SSD | 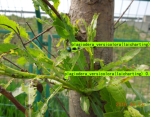 | 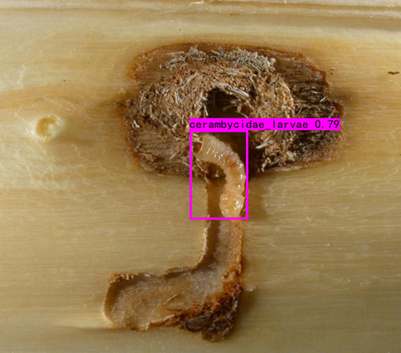 | 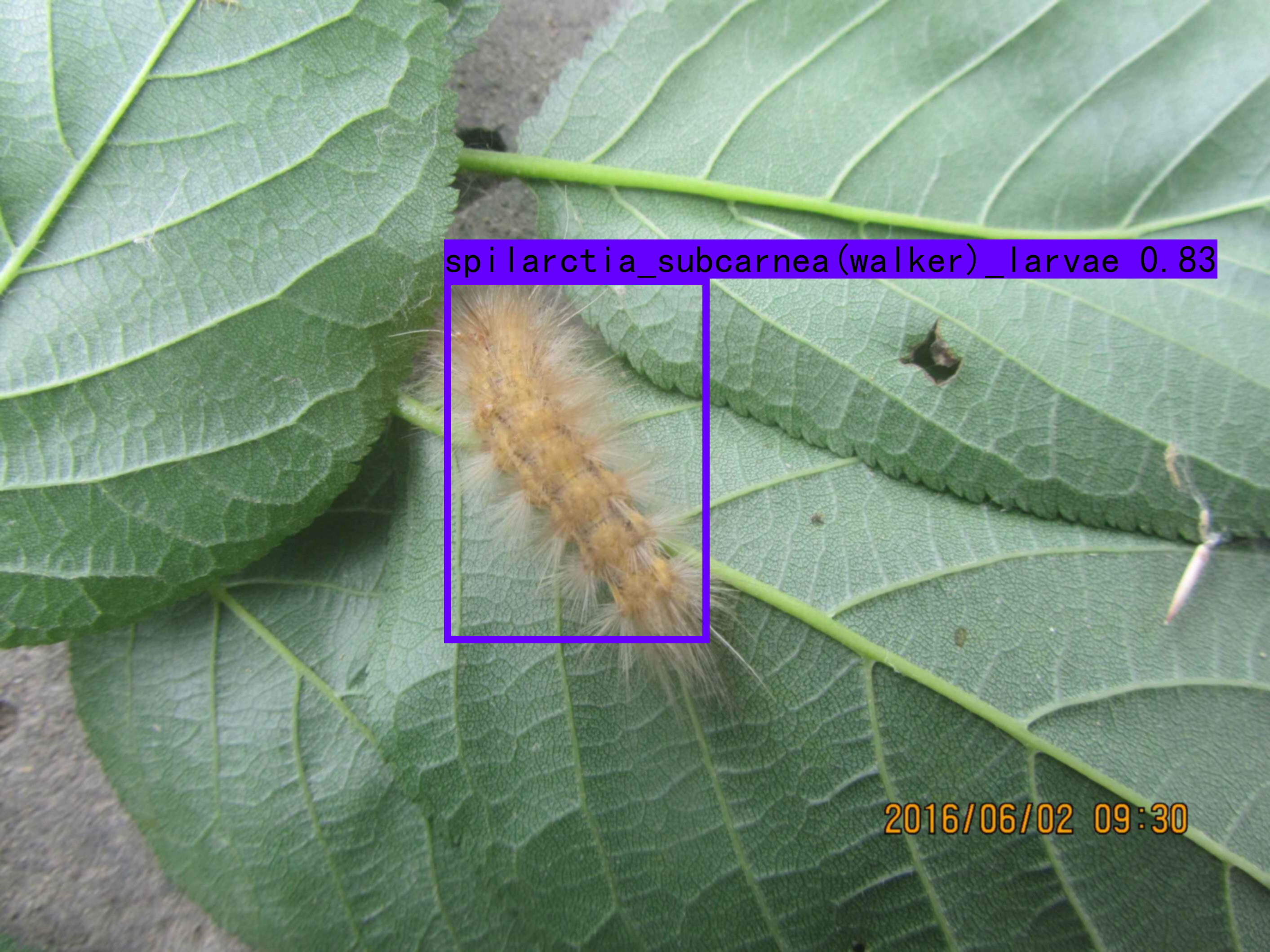 | 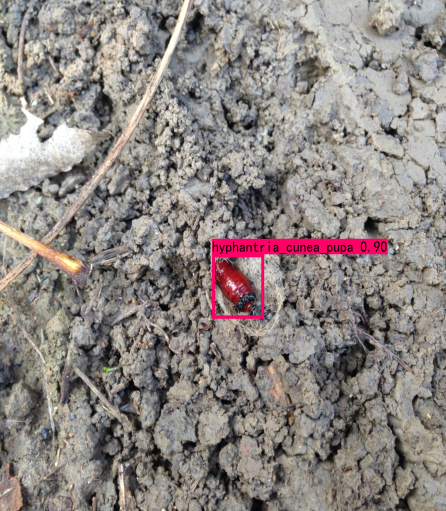 | 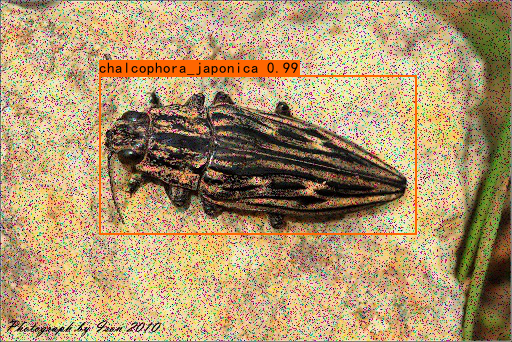 | 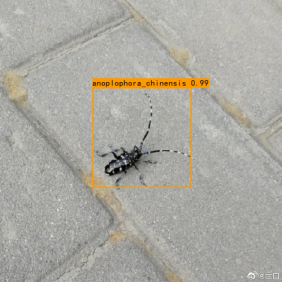 |
| Faster R-CNN | 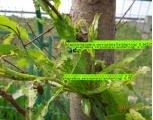 | 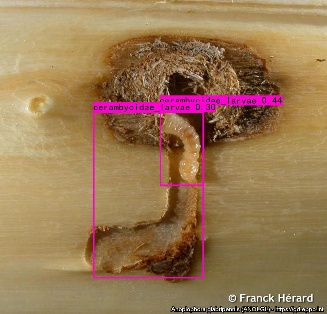 | 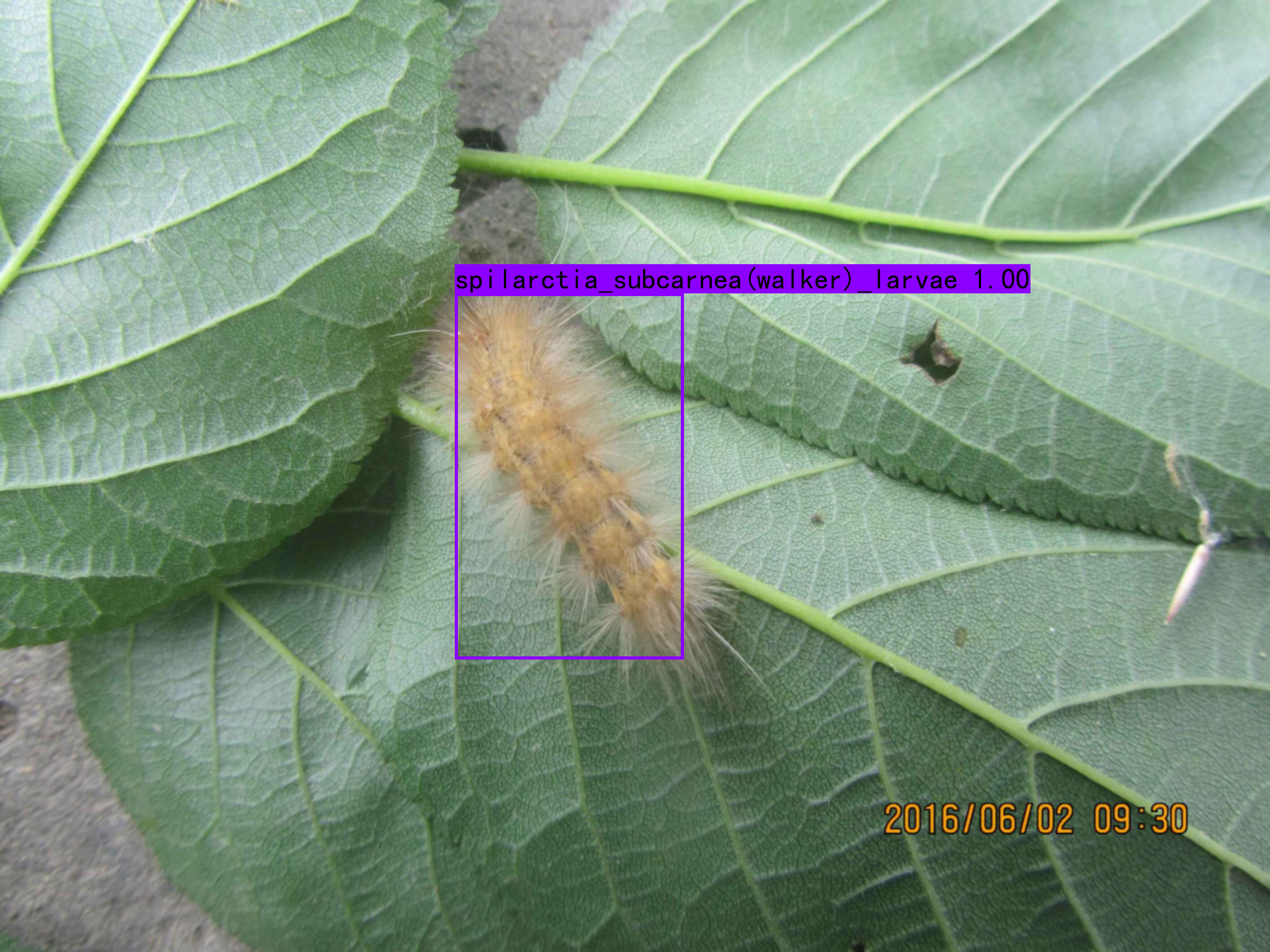 | 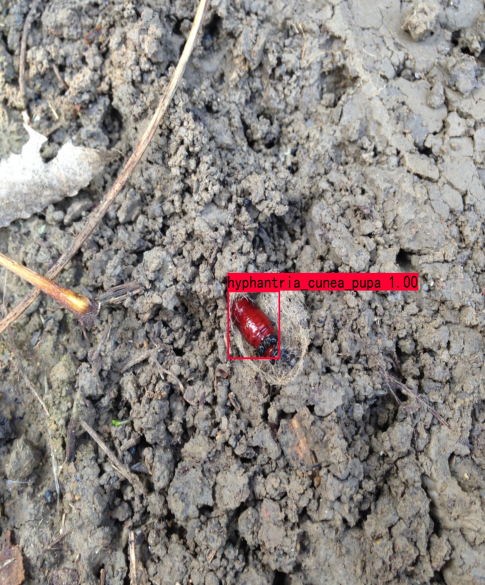 | 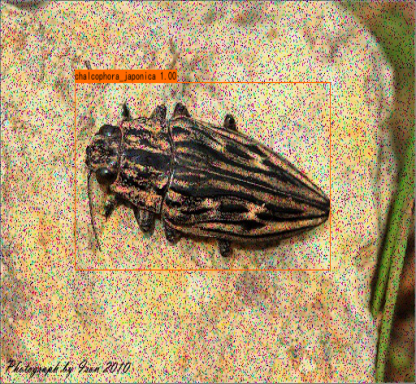 | 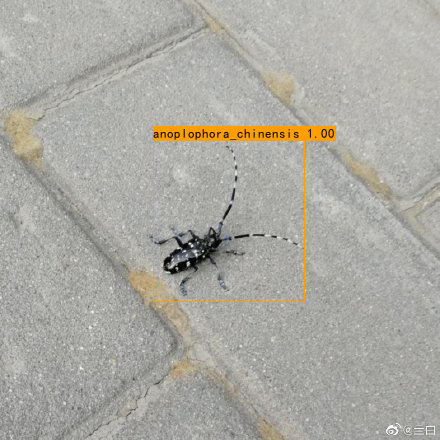 |

**Supplementary Table 1.** the test data to compare the detection results of each model.
